# Improving dermal fibroblast-to-epidermis communications and aging wound repair through extracellular vesicle-mediated delivery of *Gstm2* mRNA

**DOI:** 10.1186/s12951-024-02541-1

**Published:** 2024-06-02

**Authors:** Haiyan Wu, Zuochao Yao, Hongkun Li, Laihai Zhang, Yuying Zhao, Yongwei Li, Yating Wu, Zhenchun Zhang, Jiali Xie, Feixue Ding, Hongming Zhu

**Affiliations:** 1 Institute for Regenerative Medicine & Research Center for Translational Medicine, Shanghai East Hospital, Tongji University School of Medicine, Shanghai, 200120 China; 2 Department of Plastic and Reconstructive Surgery, Shanghai East Hospital, School of Medicine, Tongji University, Shanghai, 200120 China; 3 Department of Cardiology, Changzhi Medical College Affiliated Heji Hospital, Shanxi, 046000 China; 4 Department of Cardiothoracic Surgery, Shanghai East Hospital, School of Medicine, Tongji University, Shanghai, 200092 China; 5 Department of Cardiology, Shanghai East Hospital, School of Medicine, Tongji University, Shanghai, 200092 China; 6 Department of Neurology, Shanghai East Hospital, School of Medicine, Tongji University, Shanghai, 200092 China; 7 Department of Plastic and Reconstructive Surgery, Shanghai Ninth People Hospital, School of Medicine, JiaoTong University, Shanghai, 200001 China

**Keywords:** Extracellular vesicles (EVs), Glutathione S-transferase mu 2 (GSTM2), Needle free injection, Wound healing, Nascent polypeptide-associated complex alpha subunit (NACA)

## Abstract

Skin aging is characterized by the disruption of skin homeostasis and impaired skin injury repair. Treatment of aging skin has long been limited by the unclear intervention targets and delivery techniques. Engineering extracellular vesicles (EVs) as an upgraded version of natural EVs holds great potential in regenerative medicine. In this study, we found that the expression of the critical antioxidant and detoxification gene *Gstm2* was significantly reduced in aging skin. Thus, we constructed the skin primary fibroblasts-derived EVs encapsulating *Gstm2* mRNA (EVs^*Gstm2*^), and found that EVs^*Gstm2*^ could significantly improve skin homeostasis and accelerate wound healing in aged mice. Mechanistically, we found that EVs^*Gstm2*^ alleviated oxidative stress damage of aging dermal fibroblasts by modulating mitochondrial oxidative phosphorylation, and promoted dermal fibroblasts to regulate skin epidermal cell function by paracrine secretion of Nascent Polypeptide-Associated Complex Alpha subunit (NACA). Furthermore, we confirmed that NACA is a novel skin epidermal cell protective molecule that regulates skin epidermal cell turnover through the ROS-ERK-ETS-Cyclin D pathway. Our findings demonstrate the feasibility and efficacy of EVs-mediated delivery of *Gstm2* for aged skin treatment and unveil novel roles of GSTM2 and NACA for improving aging skin.

**Graphical Abstract:**

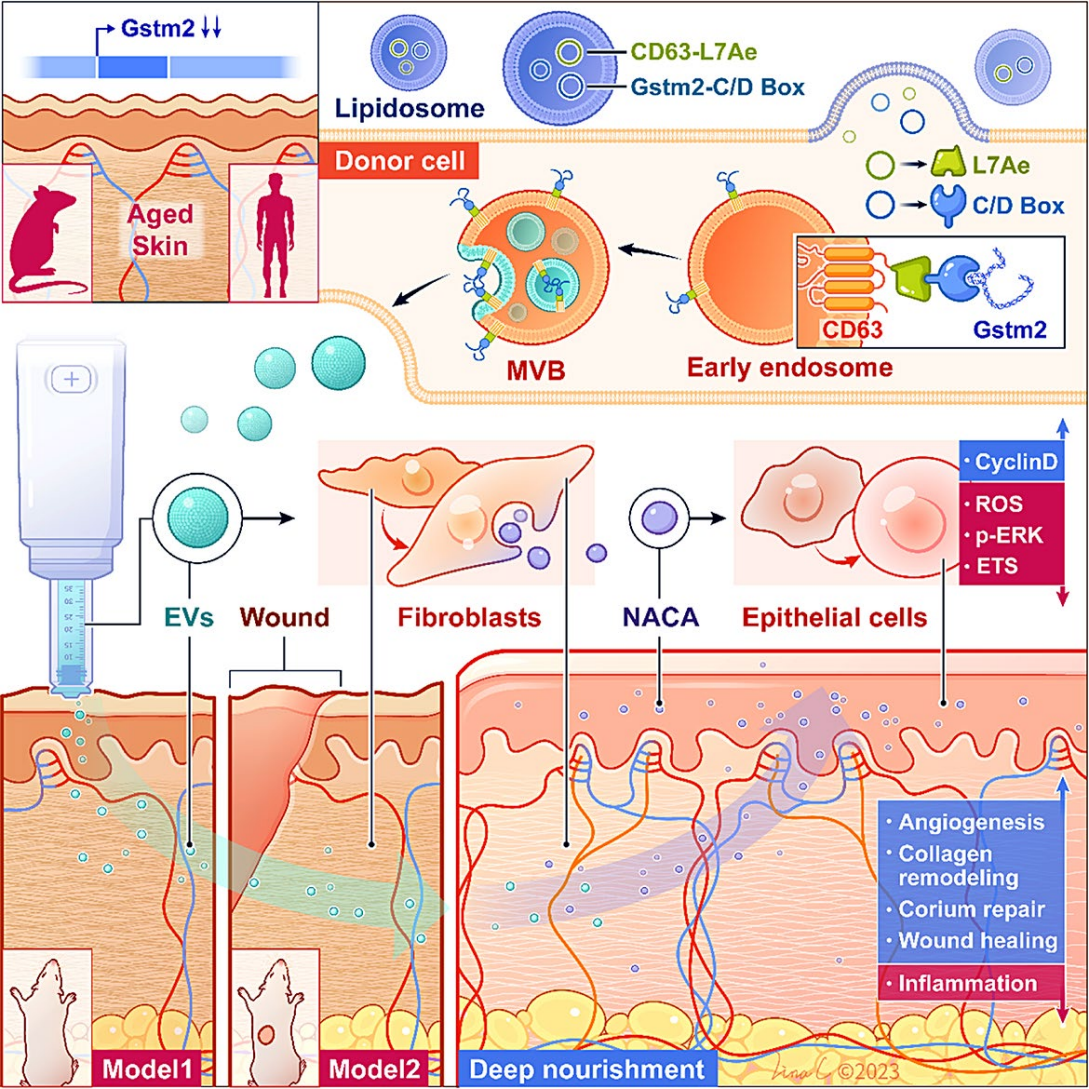

**Supplementary Information:**

The online version contains supplementary material available at 10.1186/s12951-024-02541-1.

## Introduction

Skin aging is a natural and complex biological process influenced by both intrinsic and extrinsic factors [1]. Intrinsic factors, including genetics, hormonal changes and cellular function, contribute to the process of skin aging. Mitochondria, among other cellular components, play crucial roles in maintaining skin function. There is mounting evidence indicating that oxidative stress and mitochondrial dysfunction are prominent features of ageing skin [2]. Besides, extrinsic factors, such as UV radiation, pollution, and lifestyle choices like smoking and poor nutrition, can accelerate the aging process [3]. Skin aging not only affects one’s physical appearance but can also lead to decreased self-esteem and quality of life [4]. Various treatments have been adopted to alleviate skin aging, including topical creams, optical and surgical procedures. However, these treatments have limitations, such as short-term effects, high costs, and potential side effects.

Extracellular vesicles (EVs) are small vesicles that are released by cells and contain various bioactive molecules, such as proteins, lipids, and nucleic acids [5]. They have been shown to play a crucial role in intercellular communication and tissue regeneration. Using engineering techniques to modify the surface and contents of EVs has further enhanced their bioregulatory functions compared to natural vesicles [6]. While there have been a variety of proposals for using natural and engineered EVs to treat skin wounds [7], their effectiveness remains a significant challenge due to the hostile environment in aged skin [8].

Oxidative stress occurs when there is an imbalance between the production of reactive oxygen species (ROS) and the body’s antioxidant defense system. Extensive research has demonstrated that oxidative stress plays an essential role in cell senescence and skin aging [9]. The glutathione S-transferase M2 (GSTM2) belongs to the glutathione S-transferase (GST) family, which is an important group of antioxidant and detoxifying enzymes in the human body [10]. GSTM2 binds glutathione (GSH) to ROS or other toxic substrates, forming stable and non-toxic products that protect cells from harmful stress [11]. A recent study demonstrated that EVs derived from young cells ameliorated senescent biomarkers in old cells due to their high level of GSTM2 and GST activity [12], suggesting GSTM2 holds immense potential as an anti-aging therapy. More specifically, the authors discovered that small extracellular vesicles (sEVs) derived from fibroblasts originating from young, healthy human donors (referred to as sEV-Ys) possess the capability to mitigate senescence-associated biomarkers in various aged cells obtained from older human donors, including those affected by oncogene-induced senescence and Hutchinson-Gilford progeria syndrome (HGPS). Further investigations revealed that sEV-Ys exhibit intrinsic glutathione-S-transferase (GST) activity and carry high levels of protein expression of glutathione-S-transferase mu 2 (GSTM2). Notably, the presence of GSTM2 enhances antioxidant levels and significantly improves the capacity of sEV-Ys to reverse the accumulation of reactive oxygen species (ROS) and promote an increase in the antioxidant glutathione (GSH).

Although the demonstrated ability of the *Gstm2* gene to alleviate senescence-associated biomarkers in cardiovascular, hepatic, and brain tissues, its role in aging skin tissue remains unclear. Therefore, the aim of this study is to investigate whether engineered EVs with increased levels of *Gstm2* can improve the homeostasis of aging skin tissue and promote wound healing.

The nascent-polypeptide associated complex (NAC) is a dimeric complex composed of αNAC (NACA) and βNAC subunits [13]. As a heterodimeric complex, NACA binds to newly synthesized polypeptides emerging from ribosomes [14]. Further investigations have revealed that NACA functions as a transcriptional co-activator for osteoblasts, achieved through its interaction with phosphorylated c-Jun [15]. Moreover, NACA has been shown to regulate the activity of the adaptor protein Fas-associated death domain (FADD). These findings indicate that NACA contributes to several protein complexes, playing a crucial role in cellular growth and development. In this study, we provide evidence that EVs loaded with *Gstm2* mRNA can improve skin homeostasis and enhance wound healing in aged mice. We identified a novel signal transduction pathway in which GSTM2 restores mitochondrial oxidative phosphorylation, reduces ROS levels, alleviates cell senescence, and increases the paracrine of a novel epidermal protective factor, NACA, from dermal fibroblasts. This leads to the promotion of epidermal cell turnover, providing a deep nourishment style effect.

## Results

### Gstm2 was downregulated in both mouse and human aging skin

**Fig. 1:**
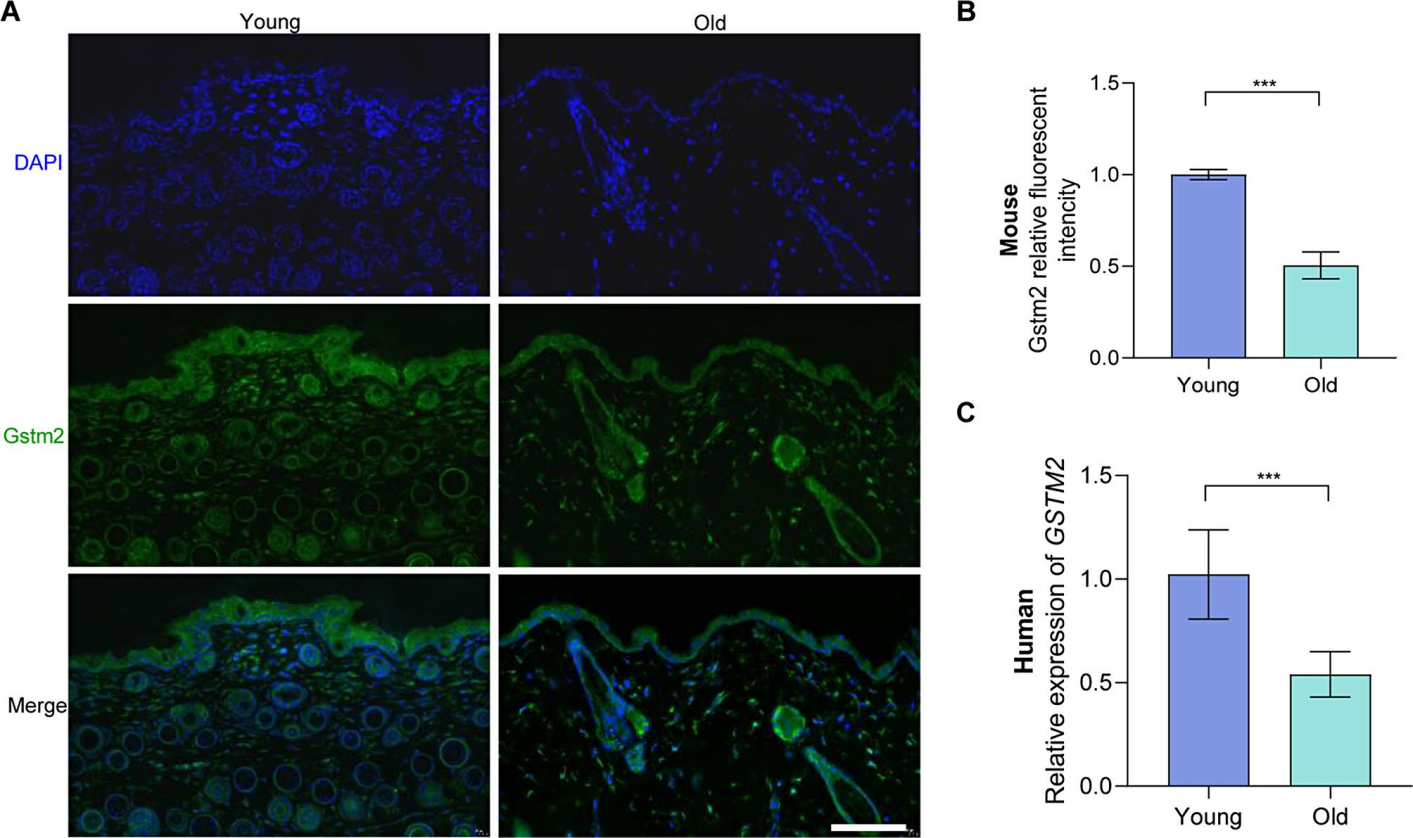
Gstm2 was downregulated in both mouse and human aging skin. (**A** and **B**) Immunofluorescence staining and quantification for Gstm2 in skin tissue of young and old mouse. Scale bar is 100 μm. (**C**) Relative *GSTM2* mRNA levels measured by qRT-PCR in skin tissue of young and old human skin. *n* = 3, ***p < 0.001

We employed immunofluorescent staining to investigate Gstm2 expression in skin tissue obtained from aged mice. The results revealed that the fluorescence intensity of Gstm2 in young mice was approximately 1.96-fold higher when compared to that in old mice (*p* < 0.001) (Fig. 1A and B). Additionally, we collected skin samples from young and old volunteers and analyzed *GSTM2* expression levels using quantitative real-time PCR (qRT-PCR). Our findings indicated that Gstm2 expression levels in young human skin samples were 1.89-fold higher than in old skin samples (*p* < 0.001) (Fig. 1C).

### Replicative senescence depressed Gstm2 expression

**Fig. 2:**
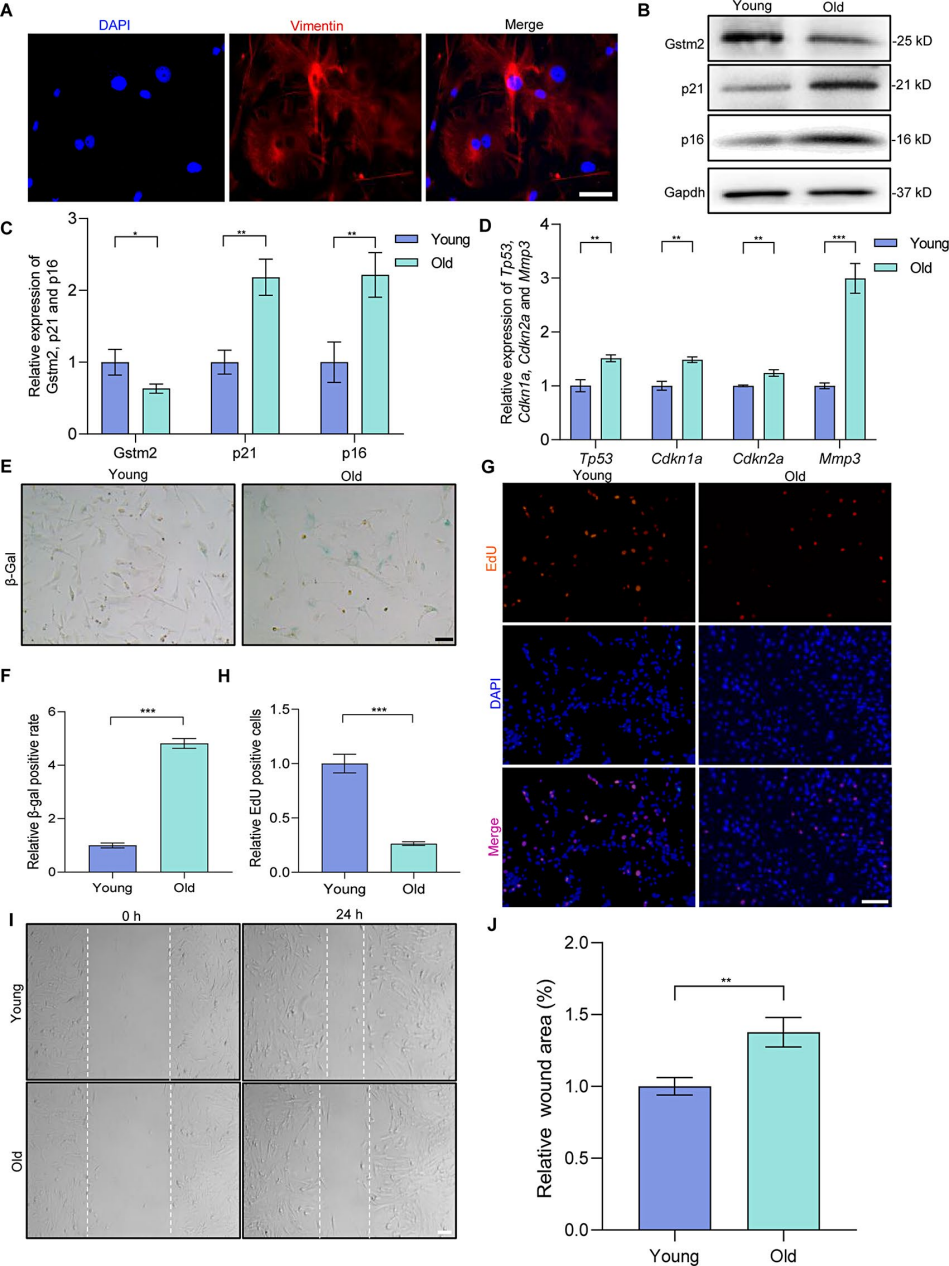
Replicative senescence depressed Gstm2 expression. (**A**) Evaluation of vimentin (red signal) expression in DFs. DAPI (blue) was used to locate the nuclei of the cells. Scale bar is 20 μm. (**B**) The expression of Gstm2 and the cellular senescence-related protein (p21 and p16) was determined by western blot in Passage 3 (P3) and Passage 10 (P10). (**C**) The quantification of the western blot results from (**B**). (**D**) The relative expression of aging-related genes (*Tp53*, *Cdkn2a*, *Cdkn1a* and *Mmp3*) was measured by qRT-PCR in P3 and P10. (**E** and **F**) SA-β-gal staining and quantification for early generation (P3) and replicative senescence (P10). Scale bar is 50 μm. (**G** and **H**) EdU staining and quantification of cell proliferation in P3 and P10. Scale bar is 100 μm. (**I** and **J**) Scratch wound healing assay and wound area statistics of P3 and P10 cells. Scale bar is 50 μm. *n* = 3, *p < 0.05, **p < 0.01, ***p < 0.001

Dermal fibroblasts (DFs) play a significant role in maintaining the flexibility and toughness of the skin. In this study, we isolated DFs from the dermal layer of the suckling mice’s skin tissue. After two days of incubation, we observed fibroblast migration from the edge of the skin tissue. By day 7, the DFs had reached up to 90% confluence, exhibiting typical fibroblast morphological characteristics, such as spindle-shaped and thin (**Fig. ****S1**). The cells were collected, seeded and confirmed by staining for Vimentin protein, a fibroblast marker (Fig. 2A). To induce replicative senescence, we subjected the dermal fibroblasts (DFs) to in vitro subculturing for up to 10 generations (p10), thus creating an in vitro aging model. Analysis of protein expression revealed elevated levels of senescence-related genes p16 (*p* < 0.01) and p21 (*p* < 0.01) in p10 compared to p3. However, the protein expression of Gstm2 exhibited a lower level in p10 compared p3 (*p* < 0.05) (Fig. 2B and C). Moreover, the mRNA level of *Tp53* (*p* < 0.01), *Cdkn1a* (*p* < 0.01), *Cdkn2a* (*p* < 0.01) and *Mmp3* (*p* < 0.001), was elevated in p10 compared to p3 (Fig. 2D). The p10 DFs exhibited enlarged, flat and irregular shape, and higher expression of SA-β-galactosidase, a marker of cellular senescence. The number of SA-β*-*Gal-positive fibroblasts was about 4.8-fold higher in p10 than in p3 (*p* < 0.001) (Fig. 2E and F). Slowed cellular proliferation and diminished migration speed were respectively confirmed by immunofluorescence staining of EdU staining and the scratch experiment in the p10 DFs. The EdU staining results showed that the p3 DFs proliferated nearly twice as fast as p10 DFs (*p* < 0.001) (Fig. 2G and H). The scratch experiment results showed that the p3 DFs migrated faster than p10 DFs (*p* < 0.01) (Fig. 2I and J). In this section, we successfully constructed an in vitro aging model using DFs.

### Genetic engineering for EVs-mediated delivery of *Gstm2*

**Fig. 3:**
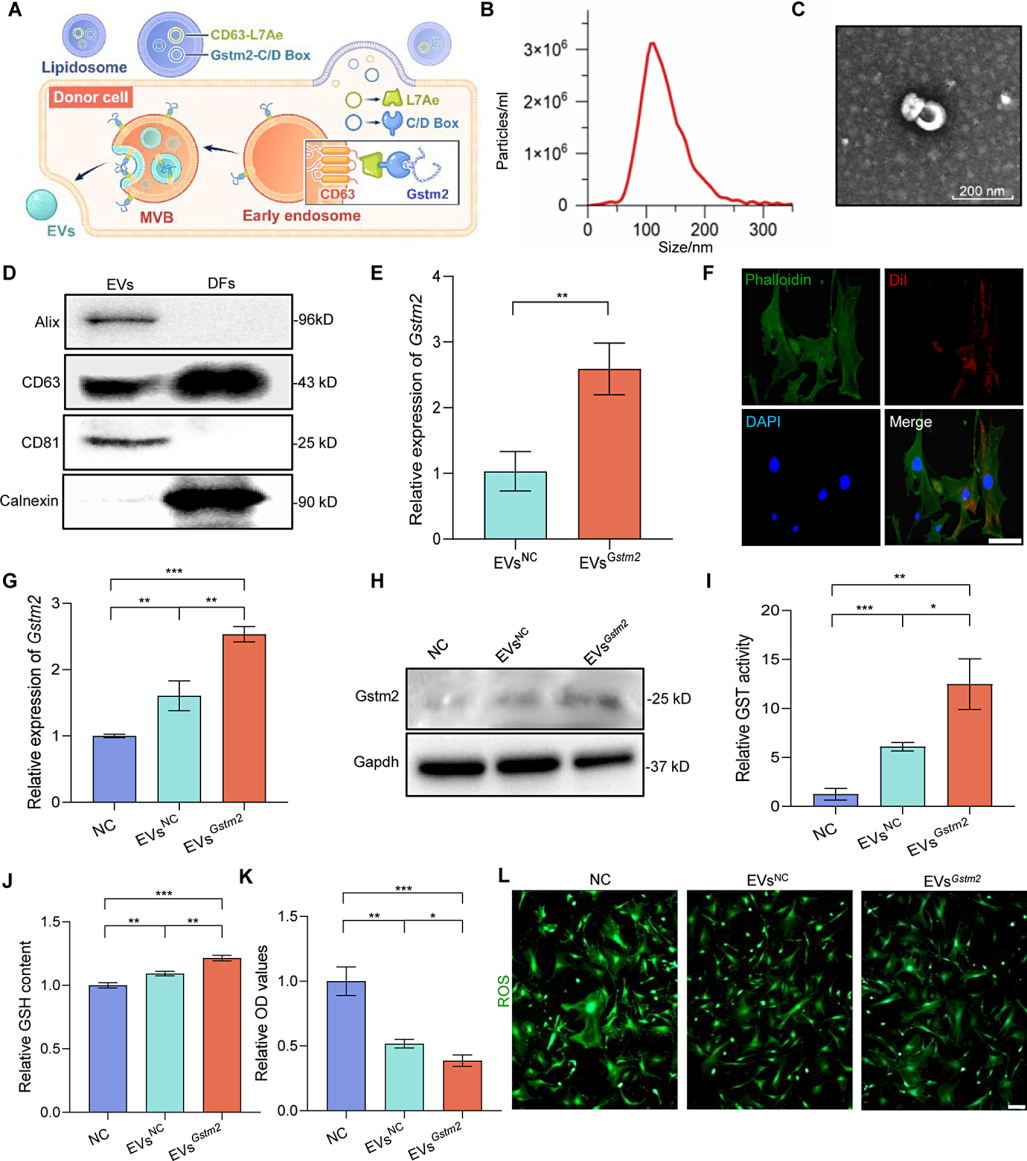
Genetic engineering for EVs-mediated delivery of *Gstm2*. (**A**) The process of EVs^*Gstm2*^ forming: the fibroblasts were transfected with the CD63-L7Ae and *Gstm2*-C/Dbox plasmids using liposomes. The CD63-L7Ae and *Gstm2*-C/Dbox constructs allow for efficient delivery of exosomal mRNA by incorporating the target gene *Gstm2* into the exosomal membrane protein CD63 through L7Ae and C/Dbox interactions. (**B**) Representative nanoparticle tracking analysis of particle diameter distribution. (**C**) Representative transmission electron microscopy (TEM) images of EVs. (**D**) Expression of EVs markers CD81, CD63 and Alix detected by western blotting. (**E**) Validation of the efficiency of *Gstm2* over expression using qRT-PCR in EVs^NC^ and EVs^*Gstm2*^. (**F**) Immunofluorescence showing internalization of EVs into DFs after 24 h of incubation. Scale bar is 20 μm. (**G**) Measurement of *Gstm2* mRNA expression using qRT-PCR in senescence DFs treated with PBS, EVs^NC^, or EVs^*Gstm2*^. (**H**) Measurement of Gstm2 protein expression using western blot in NC group, EVs^NC^ group, and EVs^*Gstm2*^ group. (**I**) Measurement of GST activity using commercial assay kit. (**J**) The GSH levels in NC group and EVs^NC^ group, EVs^*Gstm2*^ group were determined by GSH assay kit. (**K**) ROS content was evaluated by OD value. (**L**) The intracellular ROS content in NC group, EVs^NC^ group and EVs^*Gstm2*^ group was measured using the fluorescent probe DCFH-DA. Scale bar is 50 μm. *n* = 3, *p < 0.05, **p < 0.01, ***p < 0.001

Over the last decade, engineered EVs have been widely employed in various research areas, such as drug and gene delivery. In this study, we constructed genetically engineered EVs to deliver mRNA *Gstm2*. The process involved transfecting DFs using plasmids overexpressing *Gstm2*, followed by collecting and assaying supernatants from each group of cells for the extracted EVs (Fig. 3A). We purified the EVs from the DFs-conditioned medium through ultracentrifugation. We characterized the EVs by transmission electron microscopy (TEM), nanoparticle tracking analysis (NTA), and western blot analysis. The size of EVs was confirmed using NTA, which showed an average particle diameter of approximately 120 nm (Fig. 3B). TEM analysis of the acquired EVs revealed cup-shaped circular bilayers, which was the characteristic of EVs (Fig. 3C). The western blot analysis confirmed the positive expression of CD63, CD81 and Alix, and the negative expression of calnexin (Fig. 3D). Transfection efficiencies were confirmed by qRT-PCR assay, which showed that the expression of *Gstm2* in the overexpression groups was 2.6-fold higher than in the NC group (*p* < 0.01) (Fig. 3E). In addition, we labeled the EVs with CM-Dil dye (red) to confirm their efficient absorption by DFs. Co-incubation of the labeled EVs with DFs for 24 h showed that the labeled EVs co-localized with DFs, primarily at the nuclear periphery (Fig. 3F). Overall, the results demonstrated that Dil-labeled EVs were effectively internalized by DFs in vitro.

To determine whether engineered EVs could improve various cellular phenotypes associated with *Gstm2*, we co-cultured DFs with PBS, EVs^NC^ and EVs^*Gstm2*^. Firstly, we examined the *Gstm2* RNA and protein levels in senescent DFs after EVs treatment, which showed a significant increase in Gstm2 content in the EVs^*Gstm2*^ group (treated with EVs^*Gstm2*^) compared to the EVs^NC^ group (treated with EVs^NC^) and NC group (treated with PBS) (Fig. 3G and H). GSTM2 expression level is closely associated with GST activity [12]. The GST activity of old fibroblasts treated with EVs^*Gstm2*^ showed almost 2-fold than that in the EVs^NC^ group. (*p* < 0.05) (Fig. 3I). GSTM2, a cytosolic enzyme, can catalyze the conjugation of GSH to eliminate the toxic byproducts generated by ROS [11]. Our results suggest that treatment with EVs^*Gstm2*^ for 48 h showed a significant increase in GSH content (*p* < 0.01) with a slight decline in ROS (*p* < 0.05) compared to the EVs^NC^ group (Fig. 3J and L). The MDA detection results under different durations of EVs intervention are presented in Fig. S2A-B. At the 12-hour time point, neither EVs^NC^ nor EVs^*Gstm2*^ showed a significant decrease in MDA levels. However, after 24 h, EVs exhibited a notable reduction in MDA levels in both aging DFs and HaCaT cells. Specifically, compared to EVs^NC^, EVs^*Gstm2*^ demonstrated a more pronounced effect in DFs (0.729 ± 0.0322, *p* = 0.0016). Thus, EVs^*Gstm2*^ exhibited greater efficacy in reducing MDA levels and improving lipid peroxidation (Fig. S2). In this section, we successfully constructed engineered exosomes overexpressing target gene *Gstm2*, and their regulatory effects associated with *Gstm2* were initially validated on senescence DFs.

### The EVs^*Gstm2*^ improved the functions of senescent skin cells

**Fig. 4:**
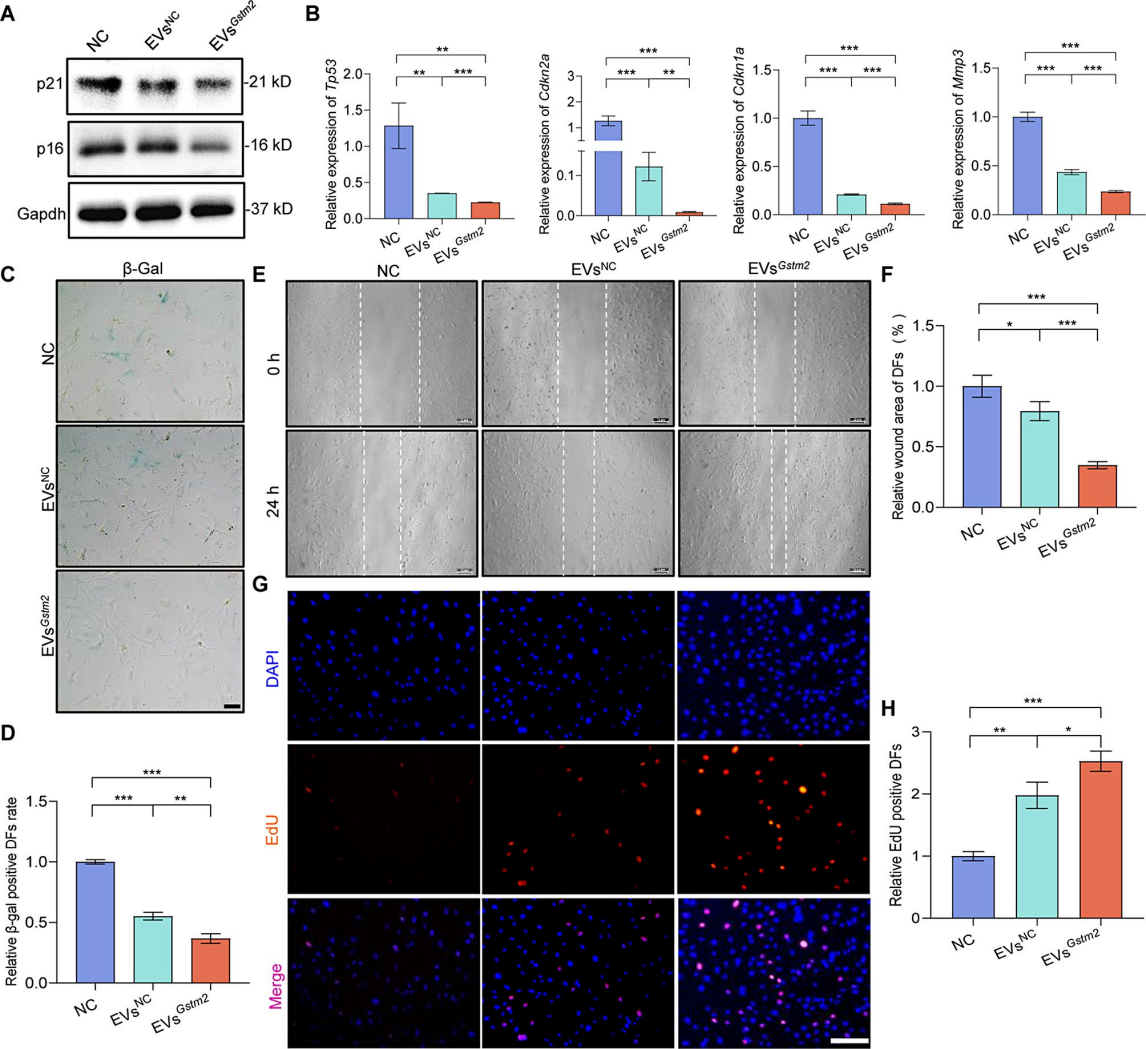
EVs^*Gstm2*^improved the functions of senescent skin cells. (**A**) The cellular senescence-related protein (p21 and p16) expression was determined by western blot in NC group, EVs^NC^ group and EVs^*Gstm2*^ group. (**B**) The relative expression of aging-related genes (*Tp53*, *Cdkn2a*, *Cdkn1a* and *Mmp3*) was measured by qRT-PCR in NC group, EVs^NC^ group and EVs^*Gstm2*^ group. (**C** and **D**) SA-β-gal staining and quantification of DFs in NC group, EVs^NC^ group and EVs^*Gstm2*^ group. Scale bar is 50 μm. (**E** and **F**) Effect of EVs^*Gstm2*^ on senescence DFs was examined using scratch wound healing assay. Scale bar is 50 μm. (**G** and **H**) EdU staining and quantification of DFs proliferation in NC group, EVs^NC^ group and EVs^*Gstm2*^ group. Scale bar is 100 μm. *n* = 3, *p < 0.05, **p < 0.01, ***p < 0.001

Dermal fibroblasts and keratinocytes are the main structural cells comprising the skin. To assess the potential of engineered EVs in improving the function of these cells, we co-cultured senescent DFs and HaCaT with PBS, EVs^NC^ or EVs^*Gstm2*^. Following treatment, we examined the expression of aging-related indicators in DFs, including senescence-related genes (*Tp53*, *Cdkn1a*, *Cdkn2a* and *Mmp3*) and proteins (p16, p21). Our findings revealed that the EVs^*Gstm2*^ group exhibited the maximum reversal of the senescence-related indicators, followed by the EVs^NC^ group and the control group (NC) (Fig. 4A and B). We used β-Gal staining to evaluate the senescence of cells in each group and observed a significant decrease in the number of β-Gal positive cells in the EVs^NC^ group and EVs^*Gstm2*^ group, with reductions of 45% (*p* < 0.001) and 63% (*p* < 0.001), respectively, compared to the NC group (Fig. 4C and D). Cell function experiments, including the scratch experiment and EdU staining, revealed that EVs^*Gstm2*^ could enhance the migration and proliferation ability of senescent DFs by about 50% (*p* < 0.001) and 32% (*p* < 0.05), respectively, compared to EVs^NC^ (Fig. 4E and H). Furthermore, we observed that treatment with EVs^*Gstm2*^ improved the proliferation and migration ability of HaCaT cells (Fig. S4 and S4). These results suggest that EVs^*Gstm2*^ can exert an anti-aging effect on senescent DFs and improve the functions of HaCaT cells in vitro.

### EVs^*Gstm2*^ modulated skin homeostasis in aged mice

**Fig. 5:**
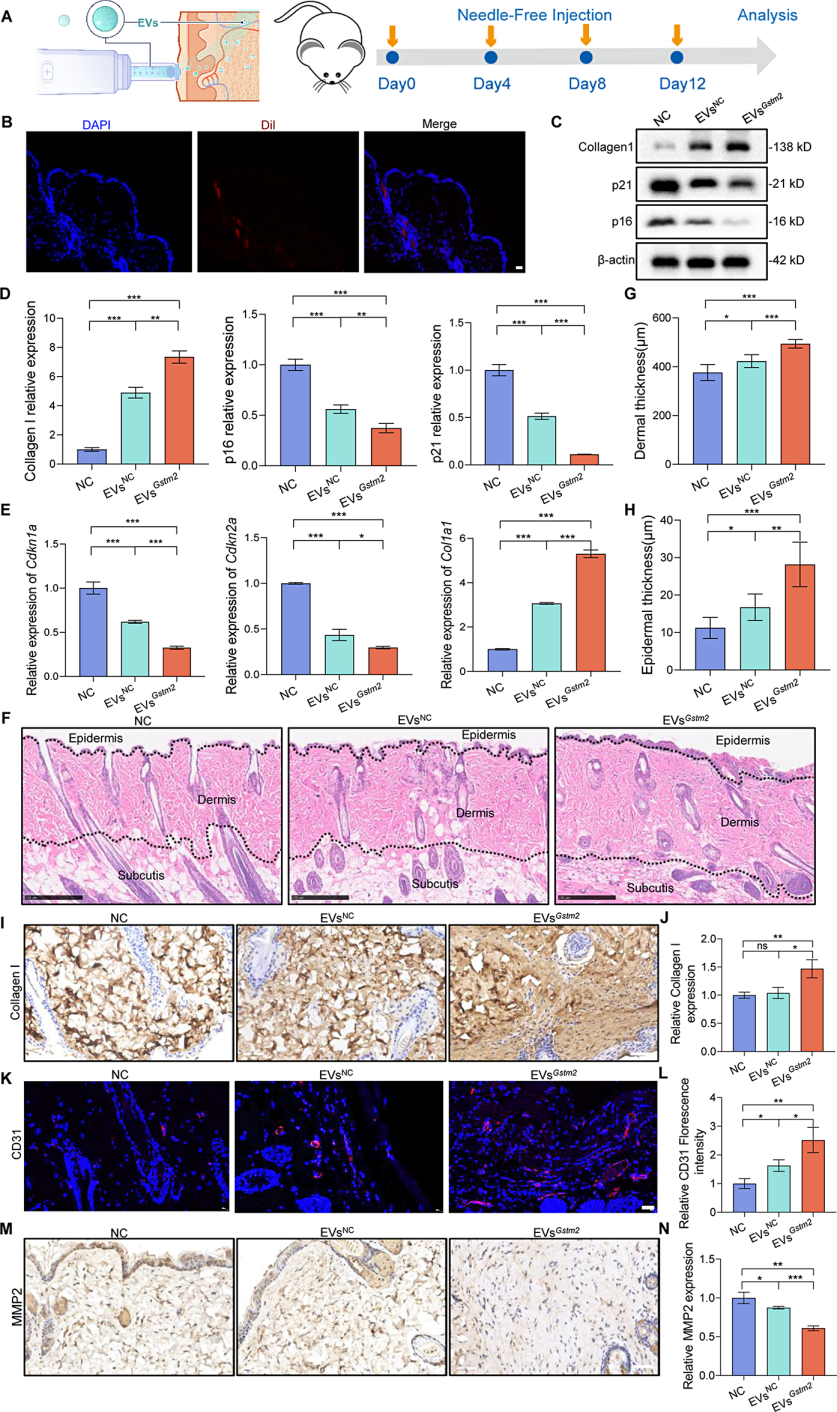
EVs^*Gstm2*^ modulated skin homeostasis in aged mice. (**A**) Schematic illustration of by needle-free injection treatment for aging mice. (**B**) Immunofluorescence staining of mouse skin tissue treated with Dil-labeled EVs. Scale bar is 50 μm. (**C**) Western blot analysis of Collagen I, p21 and p16 in skin samples. (**D**) The quantification of the western blot results from (**C**) using image J software. (**E**) The measurement of *Cdkn2a*, *Cdkn1a* and *Col1a1* expression in skin tissue by qRT-PCR. (**F**-**H**) H&E staining, dermal thickness and epidermal thickness analysis of aging mouse skin tissue. Scale bar is 250 μm. (**I**) Representative photographs of immunohistochemistry staining of Collagen I in skin tissue. Scale bar is 250 μm. (**J**) Statistical analysis on the relative expression of Collagen I in skin tissue. (**K**) Representative images of CD31 immunofluorescence staining of skin tissue. Scale bar is 20 μm. (**L**) Statistical data on the relative CD31 expression in the different groups. (**M**-**N**) Immunohistochemical staining and statistical data for MMP2. Scale bar is 50 μm. *n* = 3, *p < 0.05, **p < 0.01, ***p < 0.001

In order to evaluate the therapeutic effects of EVs^*Gstm2*^ in vivo, we injected PBS, EVs^NC^ and EVs^*Gstm2*^ into the back of 12-month-old mice using a needle-free injection. EVs with fluorescent moieties were found to be significantly enriched in dermal tissue (Fig. 5A and B). We further evaluated the EV biodistribution in mice after needle-free injection, microneedle injection, and subcutaneous injection using small animals living optical imaging. The result suggested that needle-free injection not only offer advantages over microneedle injection, but also represent an improvement over subcutaneous injection (Fig. S5). We injected 2 mL 100 µg/mL EVs^NC^, EVs^*Gstm2*^ or PBS into the back skin of the mice using needle-free injection for four times every other four days. On the 13th day of the experiment, skin tissues were collected for histology analysis and biochemical experiments, as shown in Fig. 5A. Initially, we used qRT-PCR and western blot to measure p21, p16 and Collagen I mRNA and protein levels, respectively. The levels of p21 and p16 were found to be significantly decreased in the EVs^*Gstm2*^-treated group, as demonstrated in Fig. 5C and E. Subsequently, skin tissues from each treatment group were harvested, fixed, sectioned, and subjected to staining with hematoxylin–eosin (H&E) staining as well as immunohistochemical staining for Collagen I, CD31 and MMP2 [16]. The structure of the skin was examined microscopically using H&E staining, and epidermal and dermal thickness were measured using ImageJ software. The results showed that the thickness of the dermis and epidermis increased by approximately 71 μm (*p* < 0.001) and 12 μm (*p* < 0.01), respectively, after EVs^*Gstm2*^ treatment, compared to the EVs^NC^ group (Fig. 5F and H). Collagen type I is the major collagen in normal human skin [17], and its elevated expression is a predominant characteristic in some anti-aging products [18]. For collagen regeneration, the expression of collagen in the EVs^*Gstm2*^ group was increased by 40% compared to the EVs^NC^ group (*p* < 0.05) and NC group (*p* < 0.01). No significant differences were observed between the EVs^NC^ and NC groups (*p* > 0.05), as shown in Fig. 5I and J. As aging occurs, both angiogenesis and arteriogenesis can be attenuated [19]. Therefore, the neovascularization of aging skin tissues was detected by immunofluorescence staining (IF) of endothelial marker CD31. The results showed that the fluorescence intensity in the EVs^*Gstm2*^ groups was about 1.6 times higher than in the EVs^NC^ group (*p* < 0.05), as demonstrated in Fig. 5K and L. The MMP family is known to play a key role in skin aging [20]. We checked the substrates of MMP2, which can disassemble extracellular matrix and are known to accumulate during aging [21]. As a result, the expression level of MMP2 was highest in group NC, followed by group EVs^NC^, while the expression level was lowest in group EVs^*Gstm2*^ (Fig. 5M and N). We also assessed the changes in other senescence-related gene prognostic indices such as Colony Stimulating Factor 3 (*Csf3*), C-X-C Motif Chemokine Ligand 9(*Cxcl9*), C-X-C Motif Chemokine Ligand 12(*Cxcl12*) and Interleukin-17 (*Il-17*) in each group, and found that these indicators were greatly reduced when senescent DFs were treated with EVs^*Gstm2*^ (Fig. S6). Therefore, we conclude that the EVs^*Gstm2*^ can exert an anti-senescent effect on the skin of natural aging mice.

### EVs^*Gstm2*^ accelerated wound healing in aged mice

**Fig. 6:**
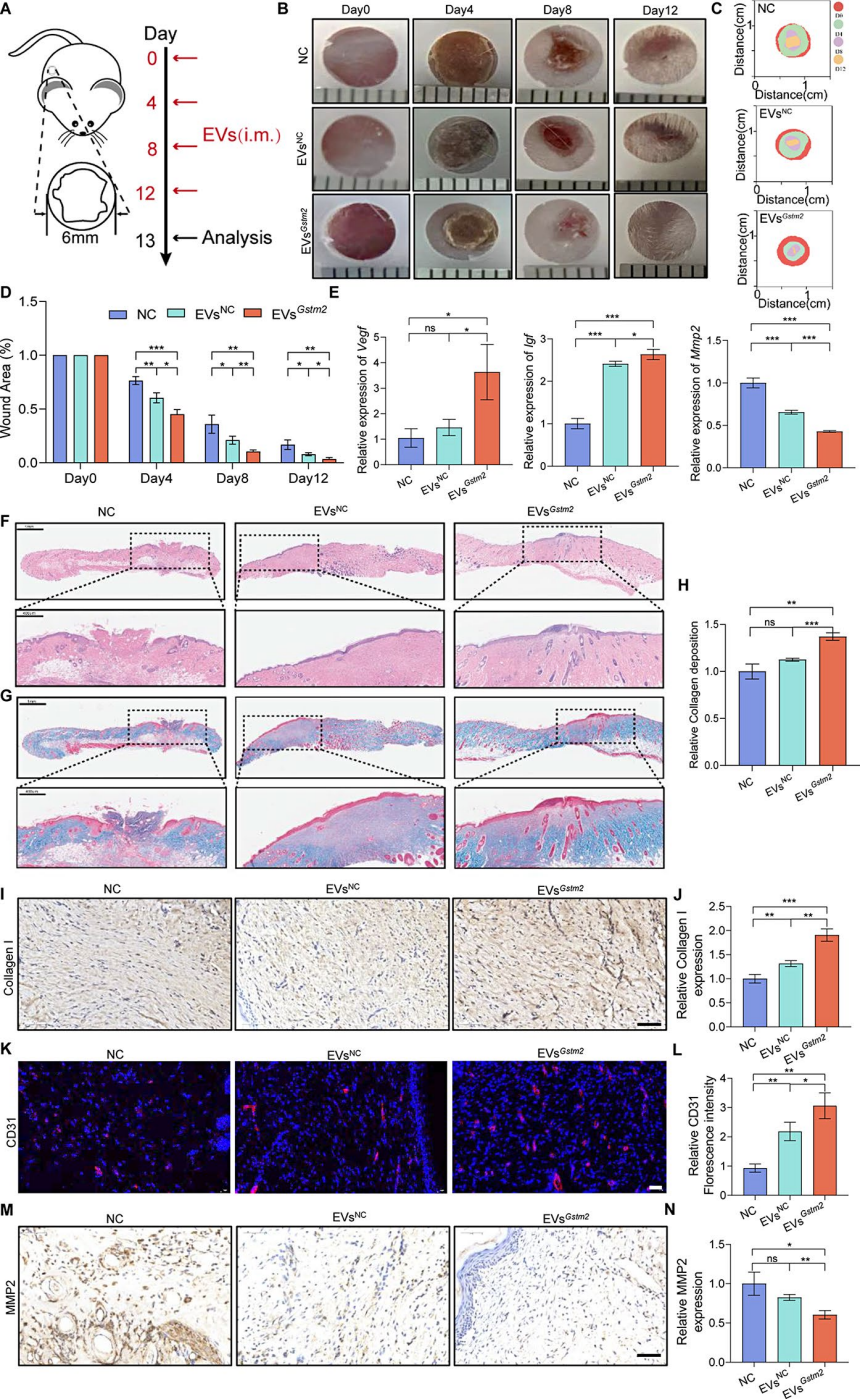
EVs^*Gstm2*^ accelerated wound healing in aged mice. (**A**) Schematic diagram of the establishment and treatment of the aging mouse wound model. (**B**) Photographs showing the morphology of skin wounds at each time point after treatment. (**C**) The simulation diagram showing the morphology of skin wound after treatment. (**D**) Analysis of the wound area in each group at each time point. (**E**) mRNA expression of prognostic indicators (*Vegf*, *Igf* and *Mmp2*) during wound healing. (**F**) Representative H&E staining results of the wound tissue in each group on day 12. (**G**) Representative Masson staining of the wound tissue in each group on day 12. (**H**) The collagen deposition in the skin tissue of the mice was quantified using Masson’s trichrome staining. (**I**-**J**) Representative immunohistochemical staining and statistical data on the relative expression of Collagen I. Scale bar is 50 μm. (**K**-**L**) CD31 immunofluorescence staining and statistical data on the relative CD31 expression of skin tissue of the different groups. Scale bar is 20 μm. (**M**-**N**) Immunohistochemical staining and statistical data for MMP2. Scale bar is 50 μm. *n* = 3, *p < 0.05, **p < 0.01, ***p < 0.001

Based on the aforementioned results, EVs^*Gstm2*^ showed a robust anti-aging effect. As reported in the literature, skin senescence can result in delayed wound healing [22]. Therefore, we constructed a skin wound healing model in natural aging mice. Firstly, we created a standardized round full-thickness wound on the back of each mouse. Next, we resuspended EVs^NC^ and EVs^*Gstm2*^ in PBS and subcutaneously injected 2 mL of 100 µg/mL EVs^NC^, EVs^*Gstm2*^ or PBS into the wound every four days for 12 days (Fig. 6A). We compared the therapeutic efficacy of these treatments with a control group (NC group). The EVs^*Gstm2*^ group demonstrated enhanced wound contraction compared to the EVs^NC^ and NC groups. Based on photographic records, we plotted a simulation map of wound closure (Fig. 6B and C). On day 12, the wounds of mice treated with EVs^*Gstm2*^ had almost completely healed, with a wound closure rate of 97%, which was significantly higher than those of the NC group (87%) and EVs^NC^ group (93%) (Fig. 6D). We then collected the skin tissue samples and assessed the changes in prognostic indicators, such as Vascular endothelial growth factor (*Vegf*) and insulin-like growth factors (*Igf*), in each group. We found that these indicators were greatly elevated when senescent DFs were treated with EVs^*Gstm2*^ (Fig. 6E). Next, we histologically analyzed the treatment effects of EVs^NC^, EVs^*Gstm2*^ and PBS on skin wound healing. We stained different treated skin tissue sections with H&E on day 13. The wounds treated with EVs^*Gstm2*^ showed obvious re-epithelization and almost no inflammation compared to those treated with EVs^NC^ and PBS (Fig. 6F). Collagen deposition is a critical factor in assessing skin strength and appearance [23]. Therefore, we stained wound tissue from day 13 with Masson staining. We observed that the EVs^*Gstm2*^ group had the highest collagen deposition density, which is 1.21-fold higher than that of the NC group (*p* < 0.01) and 1.37-fold higher than that of the EVs^NC^ group (*p* < 0.001) (Fig. 6G and H). New ECM proteins, such as collagen I, can strengthen the repaired tissue during the remodeling process [24]. In our study, immunohistochemical staining demonstrated that collagen I expression was the highest in the EVs^*Gstm2*^ group compared to the EVs^NC^ group (*p* < 0.01) and NC group (*p* < 0.001) (Fig. 6I and J). Additionally, we observed that collagen I was arranged regularly and robustly in the EVs^*Gstm2*^ group (Fig. S7). Regenerated blood vessels in wounds provide the nutrition required for wound granulation tissue and new keratinocytes, which play a significant role in wound healing. Therefore, we detected wound neovascularization using immunofluorescence staining (IF) of the endothelial marker CD31. From the IF results, we found that the CD31 expression in the EVs^*Gstm2*^ groups was approximately 1.5-fold higher than that in the EVs^NC^ group (*p* < 0.05) and 3.1-fold higher than that in the NC group (*p* < 0.01) (Fig. 6K and L). Numerous studies have implicated MMP2 in wound healing and tissue remodeling [25, 26]. From the results of immunohistochemical staining and Image J analysis, we observed that the expression of MMP2 in the EVs^*Gstm2*^group was lower (*p* < 0.01) (0.75-fold) than that in the EVs^NC^ group and lower (*p* < 0.05) (0.6-fold) than NC group (Fig. 6M and N). We also examined the mRNA expression levels of *Mmp2* and came to a similar conclusion (Fig. 6E). In conclusion, our results demonstrate that EVs^*Gstm2*^ can effectively accelerate wound healing in naturally aging mice.

### EVs^*Gstm2*^ promoted the mitochondrial oxidative phosphorylation in aged fibroblasts

**Fig. 7:**
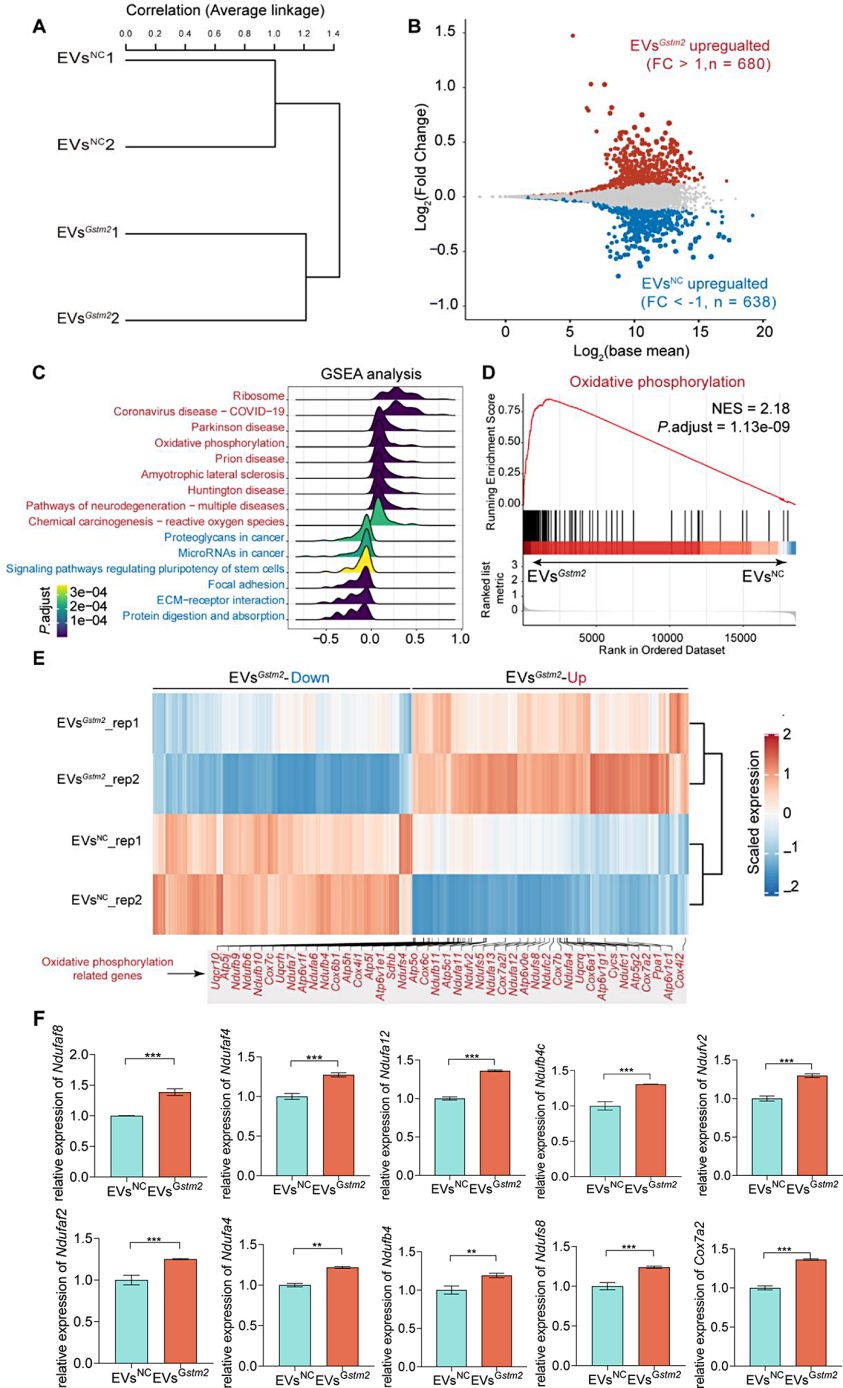
EVs^*Gstm2*^ promoted the mitochondrial oxidative phosphorylation in aged fibroblast. (**A**) The correlation among the RNA-seq samples. (**B**) Volcano plot showed the differentially expressed genes between the EVs^*Gstm2*^ and EVs^*NC*^ groups. (**C**) GSEA analysis of the activated pathways in EVs^*Gstm2*^ and EVs^*NC*^ groups. (**D**) GSEA analysis indicated that oxidative phosphorylation was activated in EVs^*Gstm2*^ group. (**E**) Heatmap showing the expression of oxidative phosphorylation-related genes. (**F**) qRT-PCR validations of the expression of oxidative phosphorylation-related genes in the groups. *n* = 3, **p < 0.01, ***p < 0.001

In order to investigate the mechanism underlying the anti-senescence effects of EVs^*Gstm2*^, we performed RNA-seq on cells treated with EVs^*NC*^ or EVs^*Gstm2*^. The biological duplicates were highly correlated, as shown in Fig. 7A. Treatment with the EVs^*Gstm2*^ resulted in 1318 differentially expressed genes (DEGs), with 680 upregulated and 639 downregulated (Fig. 7B). Gene Set Enrichment Analysis (GSEA) revealed that ribosome and oxidative phosphorylation were upregulated in EVs^*Gstm2*^ group, while focal adhesion and ECM-receptor interaction were down-regulated in the EVs^*Gstm2*^ group (Fig. 7C). Among these pathways, oxidative phosphorylation has been reported to be associated with cell senescence. We observed the activation of several oxidative phosphorylation-related genes in the EVs^*Gstm2*^ group (Fig. 7D and E). Furthermore, qRT-PCR analysis of the oxidative phosphorylation-related genes validated our observations (Fig. 7F). Taken together, our results suggest that EVs^*Gstm2*^ can improve mitochondrial oxidative phosphorylation and ameliorate cell senescence.

### EVs^*Gstm2*^ treated fibroblasts improve epidermis cell turnover through paracrine

**Fig. 8:**
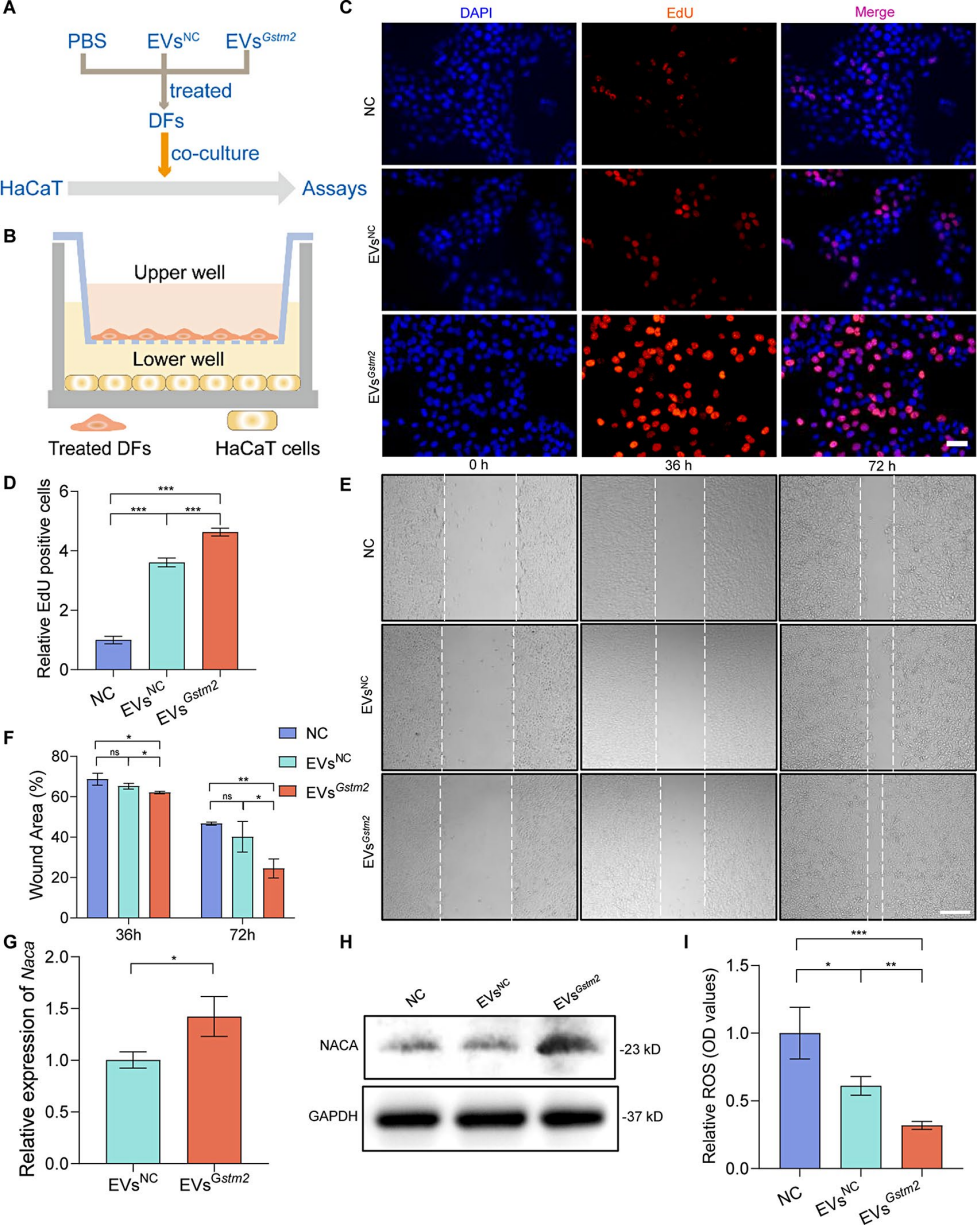
EVs^*Gstm2*^ treated fibroblasts improve epidermis cell turnover through paracrine. (**A**) A flow chart depicting the co-culturing method: the HaCaT cells co-cultured with DFs treated with PBS, EVs^*NC*^, or EVs^*Gstm2*^, followed by multiple assays. (**B**) A schematic diagram of transwell-based co-cultures system of HaCaT cells and treated DFs. (**C** and **D**) EdU staining and quantification of cell proliferation of each group. The scale bar is 50 μm. (**E** and **F**) The migration capability of HaCaT cells was assessed by scratch assay. The scale bar is 200 μm. (**G**) The expresstion of the *Naca* gene, determined by RNA-seq, was confirmed by qRT-PCR. (**H**) Western blot analysis was used to determine the expression levels of NACA. (**I**) ROS content was evaluated by OD value in the NC group, EVs^NC^ group and EVs^*Gstm2*^ group. *n* = 3, *p < 0.05, **p < 0.01, ***p < 0.001

Upon examining the pathomorphological images produced by H&E staining (Fig. 5F), we surprisingly discovered a marked therapeutic effect in the epidermis layer (Fig. 5H). As we previously demonstrated, EVs ^*Gstm2*^ act on fibroblasts in the dermis layer through needle-free injection. Therefore, we hypothesized that the function of keratinocytes could be enhanced by fibroblasts treated with EVs^*Gstm2*^ via paracrine signaling. To investigate this, we constructed a co-culturing model of DFs treated with PBS, EVs^NC^ or EVs^*Gstm2*^ and HaCaT cells (NC group, EVs^NC^ group, or EVs^*Gstm2*^ group) (Fig. 8A and B). We observed a significant improvement in the proliferation of HaCaT cells in the EVs^*Gstm2*^ group (*p* < 0.001, 4.6-fold increase) compared to the NC group and in the EVs^NC^ group (*p* < 0.001, 3.6-fold increase) compared with NC group (Fig. 8C and D). Furthermore, the migration ability of HaCaT cells in the EVs^*Gstm2*^ group was also elevated (1.45-fold increase, *p* < 0.001) compared to the NC group at 72 h (Fig. 8E and F). Interestingly, we observed filamentous cytoskeletons in HaCaT cells co-cultured with fibroblasts treated by EVs^*Gstm2*^ (Fig. S8). To investigate the mechanism behind the fibroblast influence on keratinocytes, we focused on RNA-Seq data and identified the *Naca* gene, which was significantly upregulated (1.4-fold increase, *p* < 0.05) in EVs^*Gstm2*^ group than in the EVs^NC^ group (Fig. 8G). Additionally, NACA protein expression levels were significantly increased in the HaCaT cells of the EVs^*Gstm2*^ group compared to those in the EVs^NC^ group (Fig. 8H). During aging, the production of ROS increases, reducing the cellular antioxidant status and resulting in oxidative stress, with subsequent cellular stress responses [27]. Oxidative stress is a crucial factor in cellular senescence. NACA is a kind of effective antioxidant which can regulate intracellular ROS levels [28]. These experimental results demonstrate that the ROS expression of HaCaT cells in the EVs^*Gstm2*^ group can be reduced by 50% (*p* < 0.01) compared with HaCaT cells in the EVs^NC^ group (Fig. 8I). Consequently, we suggest that the function of HaCaT cells is improved by fibroblasts treated with EVs^*Gstm2*^ via a secretory protein NACA.

### NACA derived from fibroblast regulated the epidermis cell renewal through ROS-ERK-ETS-Cyclin D pathway

**Fig. 9:**
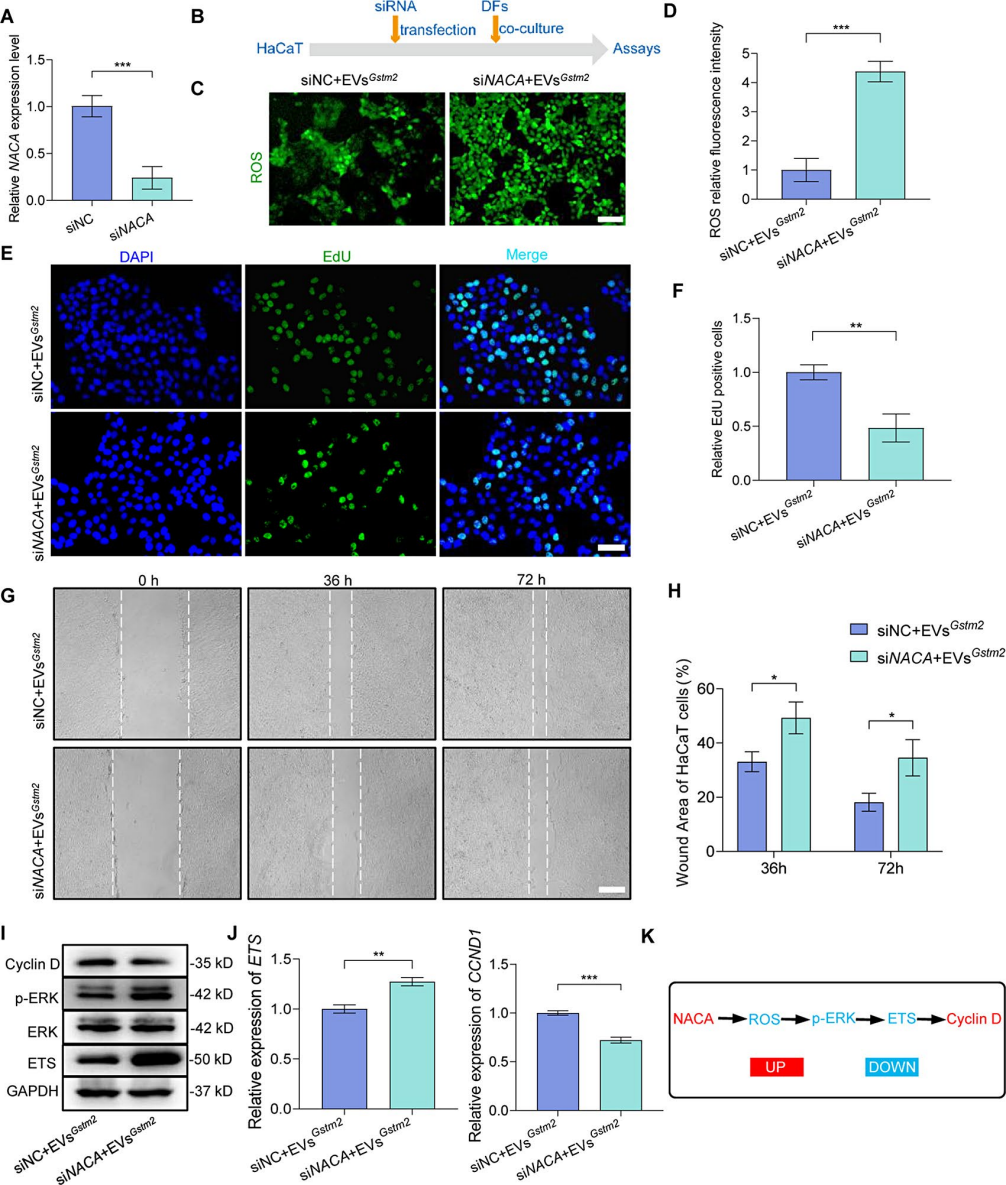
NACA derived from fibroblast regulated the epidermis cell renewal through ROS-ERK-ETS-CyclinD pathway. (**A**) The siRNA-mediated knockdown of NACA. (**B**) A flow chart describing the co-culturing method: HaCaT cells with or without Naca knockdown (siNC or siNACA) co-cultured with EVs^*Gstm2*^ treated DFs, followed by multiple assays. (**C**-**D**) The intracellular ROS content of HaCaT cells was measured using the fluorescent probe DCFH-DA. The scale bar is 50 μm. (**E**-**F**) The proliferation of HaCaT cells was investigated by EdU staining. The scale bar is 50 μm. (**G**-**H**) The migration of HaCaT cells was measured by scratch assay. The scale bar is 200 μm. (**I**) Western blot analysis was used to determine the levels of ERK, p-ERK, ETS and Cyclin D in HaCaT cells. (**J**) qRT-PCR analysis was used to determine the expression levels of *ETS* and *CCND1* in HaCaT cells. (**K**) A brief schema of molecular mechanisms of NACA regulates epidermis cells turnover. *n* = 3, *p < 0.05, **p < 0.01, ***p < 0.001

To further explore the mechanism underlying fibroblast-mediated improvement of HaCaT cell function, we employed small interfering RNA to target *NACA* (si*NACA*) and knock down its expression by 75% (*p* < 0.001), as shown in Fig. 9A. We then co-cultured HaCaT cells with or without *NACA* knockdown and fibroblasts treated with EVs^*Gstm2*^, resulting in two treatments: (a) HaCaT cells without knockdown co-cultured with fibroblasts treated with EVs^*Gstm2*^ (siNC + EVs^*Gstm2*^ group); and (b) HaCaT cells with knockdown co-cultured with fibroblasts treated with EVs^*Gstm2*^ (si*NACA* + EVs^*Gstm2*^ group), as illustrated in Fig. 9B. We found that the level of ROS in the si*NACA* + EVs^*Gstm2*^ group was significantly increased compared to the siNC + EVs^*Gstm2*^ group (*p* < 0.001) (Fig. 9C and D). Furthermore, we observed a 52% reduction in the proliferation capacity (*p* < 0.01) and a 24% reduction in the migration ability (*p* < 0.05) at 36 h, as well as a 21% reduction (*p* < 0.05) at 72 h in the si*NACA* + EVs^*Gstm2*^ group compared to the siNC + EVs^*Gstm2*^ group (Fig. 9E and H). ROS, phosphorylated extracellular signal-regulated kinase (p-ERK), estrogen sulfotransferase (ETS), and Cyclin D are key components of the classic ERK signaling pathway. The classical ERK signaling pathway is known to be regulated by changes in intracellular ROS levels. Additionally, this pathway plays a significantly role in modulating the expression of ETS transcription factors, which subsequently affects the activation of cyclin D1 [29]. In this study, we demonstrated that extracellular vesicle-mediated delivery of *Gstm2* mRNA activated this signal axis in epidermis cells (HaCaT cells). The levels of p-ERK and ETS, downstream of NACA, were rapidly increased upon *NACA* knockdown, which in turn inhibited Cyclin D expression (Fig. 9I). Similar results were obtained with qRT-PCR analysis at the *RNA* level. The expression levels of *ETS* in the siNC + EVs^*Gstm2*^ group was decreased (*p* < 0.01), while the expression levels of *CCND1* in the siNC + EVs^*Gstm2*^ group were significantly increased (*p* < 0.001) compared to the si*NACA* + EVs^*Gstm2*^ group (Fig. 9J). Therefore, we conclude that the function of HaCaT cells was improved by the secretory protein NACA through ERK signaling (Fig. 9K).

## Discussion

In this study, we found replicative senescence reduced GSTM2 expression in fibroblasts and aged skin. Through EVs-mediated delivery of *Gstm2*, we demonstrated that Gstm2 enhanced cellular motility in senescent cells, modulated skin homeostasis, and accelerated wound healing in aged mice. Mechanistically, treatment with EVs-*Gstm2* ameliorated cellular oxidative stress by modulating mitochondrial oxidative phosphorylation in aged fibroblasts and improved epidermis cell turnover through paracrine signaling. Furthermore, we identified NACA, a novel regulator of epidermis cell turnover, derived from fibroblasts, which acted through the ROS-ERK-ETS-Cyclin D pathway. Our findings demonstrated the feasibility and efficacy of EVs-mediated delivery of *Gstm2* for aged skin treatment and unveiled novel roles of GSTM2 and NACA for improving aged skin.

The human skin is comprised of three main layers: the epidermis, dermis, and subcutaneous tissue [30]. As the outermost layer of the skin, the epidermis directly encounters external stressors such as UV radiation, making its cells a critical target in anti-aging research. However, due to the low transdermal absorption rate of the epidermis and its rapid turnover rate, the efficiency and duration of topical drug delivery are limited, greatly hindering therapeutic efficacy [31]. In recent years, anti-aging research targeting fibroblasts in the dermis has gained more attention. As the ‘foundation’ of epidermal cells, dermal fibroblasts not only provide structural support but also dynamically regulate the structure and function of epidermal tissue through paracrine signaling. A pioneering study showed that fibroblast secretions were more effective in improving skin aging compared to stem cells [32]. It has been demonstrated that engineered EVs can be safely and effectively delivered to the dermis using a needle-free injector [33–35]. In this study, we used a needle-free injector to deliver engineered EVs to dermal fibroblasts, successfully achieving local homeostasis regulation function in mouse skin tissues.

The glutathione S-transferases (GSTs) are found ubiquitous in the animal and plant kingdom and have been identified in most of human tissues. Several human GSTs have been isolated, with distinct substrate specificities and catalytic efficiencies. Among these enzymes, GST mu (GSTM), pi (GSTP), and theta (GSTT) are the most commonly found with shared variants. The GSTM1 gene experiences a frequent polymorphism resulting in a large deletion, with up to 50% of Caucasoid individuals being homozygous for this variant (GSTM1null) [36]. As to GSTM2, prior research has revealed the regulatory effect of GSTM2 on pulmonary [37], hepatic [38], skeletal muscle [39], and cardiac function [40], with particular emphasis on its antioxidant and detoxifying properties. Moreover, studies have shown that GSTM2 regulates tumor cell metastasis and treatment sensitivity [41–43]. Reports on other functions of GSTM2 remains limited. Recently, Juan Antonio et al. found that EVs derived from young cells significantly reduced the expression of aging marker (β-Gal) in the kidney, liver, lung, and adipose tissues of aged mice. Further investigation revealed that this effect was due to the GST activity of GSTM2 carried by EVs from young donors [12]. While this study suggests the potential anti-aging capabilities of GSTM2, it is worth noting that β-Gal staining results cannot be equated with the degree of aging; cellular function is a more accurate reflection of aging level. Additionally, the natural abundance of GSTM2 in EVs from young donors is very low, making clinical translation difficult. To overcome these limitations, the present study constructed a CD63-L7Ae-C/D Box plasmid system to overexpress *Gstm2* mRNA and package it into EVs.

The Nascent Polypeptide-Associated Complex (NAC) is a highly conserved protein complex that associates with ribosomes during protein translation [44]. NAC binds to nascent polypeptides as they emerge from the ribosome and facilitates proper folding by preventing premature interactions with other proteins or cellular components. Moreover, NAC plays a critical role in targeting misfolded proteins for degradation by the proteasome, which is responsible for breaking down and recycling proteins [45]. The NAC complex is composed of two subunits, alpha and beta [46]. The alpha subunit of NAC (NACA; Hom s 2) has been identified as a c-Jun coactivator [47] and an IgE-reactive autoantigen in patients with severe forms of atopy [48]. Recent studies have shown NACA induce T-cell proliferation, monocyte activation and secretion of cytokines such as IFN-γ, IL-17, and IL-22 [49, 50]. In this study, we have discovered a novel function and mechanism of NACA in regulating skin epidermal cell turnover. Specifically, NACA downregulates intracellular ROS and its induced ERK phosphorylation, which inhibits ETS expression, thereby upregulating the expression of the cell cycle regulating molecule Cyclin D, ultimately promoting skin epidermal cell proliferation and turnover. Our results expand the current understanding of NACA function and target cells, revealing its role, source, and target cells in maintaining the homeostasis of aging skin. Furthermore, we have demonstrated that NACA not only acts as a coactivator of c-Jun [47], but also regulates the phosphorylation of another protein kinase, ERK. Since ERK is a key molecule that transmits biological signals from surface receptors to the cell nucleus, the above results suggest that NACA may participate in the process of extracellular signal transduction into the nucleus.

Our study has some potential limitations. Firstly, regarding the source of EVs, it is well known that the cell origin of EVs directly influences crucial characteristics such as cargo composition, biological activity, and targeting ability [51]. In this study, we solely utilized primary fibroblasts as the source of EVs, without comparing other cell sources such as mesenchymal stem cells, blood-derived cells, or human cell lines. The primary rationale behind this choice was to avoid the low affinity and potential immunogenicity associated with interspecies application of EVs. Future research may explore engineered EVs derived from mesenchymal stem cells to assess their effects on aging skin, aiming to investigate a more effective and clinically translatable cell source for EVs. Furthermore, in terms of molecular mechanisms, although we have demonstrated for the first time the role of NACA in improving skin aging through dermal-epidermal crosstalk, we have not explored the underlying mechanisms in depth. For instance, the distribution of NACA as a transcriptional co-activator in aging skin cells remains unknown. Which transcription factor does it interact with? Where are the binding sites located? These scientific questions remain to be addressed in future studies to gain a deeper understanding of these mechanisms.

In summary, our study highlights the potential of engineered EVs as a promising tool to treat aging skin. By encapsulating *Gstm2* mRNA within EVs (EVs^*Gstm2*^), we demonstrate significant improvements in skin homeostasis and wound healing in aged mice. Mechanistically, we found that EVs^*Gstm2*^ modulated mitochondrial oxidative phosphorylation and promoted DFs to regulate skin epidermal cell function through paracrine secretion of NACA. Moreover, we identified NACA as a novel protective molecule that regulates skin epidermal cell turnover through the ROS-ERK-ETS-Cyclin D pathway. These findings provide new insights into potential intervention targets for improving aging skin and demonstrate the feasibility and efficacy of EVs-mediated delivery of *Gstm2* for this purpose (Graphical Abstract).

## Materials and methods

### Human samples

This study was approved by a local institutional review board and ethics committee of Shanghai Ninth People’s Hospital, and it adhered to the tenets of the Declaration of Helsinki (SH9H-2019.T213-3). Human skin samples were collected from mild-to-moderate ptosis in the plastic and reconstructive surgery of Shanghai Ninth People’s Hospital (Shanghai, China) (Supplementary Table 1). All patients had signed the informed consent and agreed the collection of tissue samples for additional histological examinations. For the experiments, skin tissue was weighted, ground and added to TRIzol reagent. The total RNA in the tissues were extracted following the manufacturer’s protocols.

### Isolation and identification of dermal fibroblast

Skin tissues under the axillary of mice were harvested. In brief, full-thickness skin harvested from the axillary of newborn mice is cut into pieces, about 0.1 cm^2^. After repeated cleaning of the debris, the pieces were placed in phosphate-buffered saline (PBS) with 1 mg/mL Collagenase I (Sigma-Aldrich, SCR103) and incubated on a rotation shaker at 37°C for 40 min. Then, the digestion was terminated with a culture medium containing the serum, the culture medium and digestion were removed, and the tissue was washed twice with PBS. Next, tissue pieces were suspended in a complete medium, which consisted of DMEM/F12 with 15% fetal bovine serum (FBS, ExCell Bio, FND500) and 1% penicillin-streptomycin (Gibco, 10,378,016) and incubated at 37°C in a 5% CO_2_ humidity 95% air incubator. After at least 24 h, fibroblasts “crawl” out of the tissue fragments and attach to the bottom of the cultural flasks. When DFs were at 90% confluent, they were collected and passaged until passage three. For the identification of fibroblasts, the DFs were fluorescently stained with vimentin and DAPI (Sigma-Aldrich, D9542).

### Replicative senescence model of fibroblasts

DFs were cultured to the third passage with the conditional medium which was consisted of DMEM/F12 with 15% FBS and 1% penicillin-streptomycin and was changed every two days. Afterward, the conditional medium was adjusted to 10% FBS. Replicative cellular senescence used in this study is a common experimental aging model [52]. We build a replicative senescence model of mouse primary skin fibroblast by repeated passage to simulate the aging process of normal cells. In brief, murine DFs isolated from newborn skin were passaged at a 1:3 ratio and until P10 for the following experiments [53].

### Extraction and purification of extracellular vesicles

Extraction of cell-derived EVs using the high-speed centrifugation method has already been reported in the literature [54–56]. Briefly, exosome-depleted FBS was prepared to effectively exhaust EVs by ultracentrifugation at 100,000 g for 2 h at 4°C. The supernatant was collected and subsequently filtered by a 0.22-µm filter (Millipore). Extracellular vesicles were isolated from distinct conditioned media samples. These conditioned media were obtained by subjecting dermal fibroblasts to different transfection conditions, including non-transfected cells, cells transfected with empty vectors, and cells transfected with a *Gstm2* overexpression vector. The conditioned media were collected 48 h after transfection. DFs from the empty vector group (EVs^NC^) and *Gstm2* overexpression group (EVs^*Gstm2*^) cells were cultured in DMEM/F12 conditioned medium containing 10% EV-free FBS and 1% penicillin–streptomycin for 48 h and the conditioned medium was collected after cultivation. Thirdly, the conditioned medium was harvested for centrifugation at 300 g, 2000 g and 10,000 g for respectively 10 min, 10 min and 30 min to remove the dead cells and cell debris in the medium; subsequently, the supernatant was centrifuged at 100,000 g for approximately 2 h twice by ultracentrifugation (Beckman Coulter Optima L-90 K ultracentrifuge; Beckman Coulter, Fullerton, CA, USA). Finally, we used PBS to resuspend the required pellets, which were used immediately or stored at –80°C for further experiments.

### Characterization of EVs

The typical morphology of the collected EVs was observed using a transmission electron microscope (TEM; HT7800, Japan). EVs were added dropwise to 200-mesh grids and incubated for 10 min at room temperature, then the grids were negatively stained with 2% phosphotungstic acid for 3 min, and the remaining liquid was removed using filter paper, and the grids were analyzed under a transmission electron microscope HT7800. The EVs particle size and concentration were measured by nanoparticle tracking analysis (NTA) at VivaCell Shanghai with Zetaview-PMX120-Z (Particle Metrix, Meerbusch, Germany) and corresponding software ZetaView (version 8.04.02, Germany). Isolated EVs were appropriately diluted with 1X PBS buffer. NTA measurement was recorded and analyzed at 11 positions. The ZetaView system was calibrated with 110 nm polystyrene particles. Temperature was maintained around 23$^ \circ {\rm{C}}$ and 30$^ \circ {\rm{C}}$. Western blots were used to evaluate the expression of the EVs markers, such as Alix (1:1000, Abcam, ab275377), CD63 (1:1000, Abcam, ab216130), CD81 (1:1000, Abcam, ab109201), and Calnexin (1:1000, Abcam, ab22595).

### Transfection of DFs

For plasmid transfections, 0.8 × 10^6^ P3 DFs were seeded in 6-well plates and cultured for 24 h. Cells were transiently transfected with polyethyleneimine (PEI) (polysciences, 23966-100) using a cDNA/PEI ratio of 1:3. In the empty vector group (EVs^NC^), the transfection medium was composed of Opti-MEM™ (Gibco, 31985-070) with 2 µg pcDNA3.1-CD63-L7Ae, 2 µg pcDNA3.1-nanoluc-C/Dbox, and 12 µL PEI. In EVs^*Gstm2*^ groups, the transfection medium was composed of Opti-MEM™ with 2 µg pcDNA3.1-CD63-L7Ae, 2 µg pcDNA3.1*-Gstm2*-C/Dbox and 12µ PEI. After 6 h of incubation, the transfection medium was replaced with a complete medium. The cell culture supernatant was collected after 48 h culture, and EVs were separated by differential centrifugation as previously described.

### Measurement of GST activity

Senescent fibroblasts treated respectively by PBS, EVs^NC^, and EVs^*Gstm2*^ were carefully collected and used to measure GST activity via using a commercial GST activity assay kit (Sangon Biotech, D799612-0100) according to the instruction protocol. Briefly, senescent fibroblasts were homogenized in 500 µL of extraction solution (provided in the kit) with an ultrasonic cell crusher on ice followed by centrifugation (8000 g/min) at 4°C for 10 min. The supernatant was collected and various reagent were added in a 96-well plate in order. After a 5s incubation at room temperature and a 5 min incubation at 37°C, absorbance at 340 nm was individually recorded with a microplate reader. The GST activity of each sample was subsequently estimated.

### Determination of the cellular GSH levels

The cellular GSH content was measured using a commercially available GSH assay kit (Nanjing Jiancheng Bioengineering Institute, China). Cells were seeded to 90 cm^2^ dishes in triplicates and treated with PBS, EVs^NC^ and EVs^*Gstm2*^. After 48 h, the following operations were conducted strictly according to the manufacturer’s instructions. GSH content was determined with a spectrophotometer at 405 nm.

### Internalization of EVs

According to the manufacturer’s instructions, EVs were labeled with CM-Dil red fluorescent membrane linker dye (Beyotime, C1035) as previously described [57]. In brief, 50 µg EVs were labeled by CM-Dil stock solution (5 µL, 1 mg/mL) and incubated at room temperature for 5 min. After incubation, unbound CM-Dil was removed via ultrafiltration centrifugation at 100,000 g for 2 h at 4°C; then, the EVs were resuspended in PBS and repeatedly washed with PBS thrice. DFs were incubated with Dil-labeled EVs (50 µg/mL) for 24 h. Then, DFs were washed three times with PBS and fixed in 4% paraformaldehyde solution. The nucleus was stained by DAPI, F-actin of DFs were stained with 50 µg/mL phalloidin-FITC (YEASEN, 40736ES75), and the images of cellular uptake of EVs were captured by fluorescence microscopy.

### In vivo bio-distribution of EVs

Purified EVs (200 µg/mouse) labeled with Dil were suspended in PBS as previously described and injected with a needleless syringe on the back. The mice were treated with light shielded for 6 h and were sacrificed. Their dorsal skins were harvested, and longitudinal frozen Sect. (10 μm) were made. Sections were counter-stained with DAPI before imaging. Finally, the frozen sections were photographed with a fluorescence microscope.

### Senescence-associated β-galactosidase (SA-β-gal) staining

The expression of senescence-associated β-galactosidase (SA-β-Gal) was measured by using the SA-β-gal staining kit (Beyotime, C0602) [58]. When the cells’ confluence reached approximately 70%, they were used for experiments such as fixation and staining. After washing with PBS, fixed DFs were incubated with β-gal staining mixture overnight at 37°C. β-gal-positive cells were expressed as the ratio of β-Gal positive cells to total cells.

### RNA extraction and quantitative real-time PCR (qRT-PCR) analysis

Total RNA was extracted from DFs or HaCaT cells with Trizol universal reagent (Tiangen, Beijing) following the manufacturer’s protocol. The total RNA (500 ng) of each sample was reversely transcribed into cDNA by HiScript III RT SuperMix for qPCR (+ gDNA wiper) (Vazyme, R323-01), real-time qPCR was performed with ChamQ Universal SYBR qPCR Master Mix (Vazyme, Q711) to quantify gene expression level. The relative gene expression levels were calculated by the 2^–ΔƊCt^ method. The primer sequences used in this study are listed in Supplementary Table 2.

### Western blotting

DFs or HaCaT cells were washed three times by a cold PBS. Samples from different treatments were prepared with RIPA buffer (Beyotime, P0013B) containing 1mM phenylmethanesulfonyl fluoride (PMSF, Beyotime, ST2573) and protease inhibitor (Beyotime, P1005). Protein concentrations were measured by BCA protein assay Kit (Thermo Fisher Scientifific, 23,225). For each sample, 30 µg protein denatured was separated by SDS-PAGE and electrophoretically transferred to a PVDF membrane. The blots were blocked with 5% BSA in TBST solution for 60 min, incubated overnight with primary antibodies at 4°C and incubated with the secondary antibody for 2 h at room temperature. Finally, immunoreactive bands were detected by ECL reagents (Keygen Biotech, SQ202L). Band signal intensities were quantified using the ImageJ software (version 1.46r, USA).

### 5-Ethynyl-2’-deoxyuridine (EdU) staining

For EdU staining, EdU Staining Proliferation Kit (Beyotime, C0078S) was used. In brief, cells were seeded in a 12-well plate and EdU solution was added for 4 h incubation. Then, fixative solution and permeabilization buffer were added for 20 min. Then, reaction mix was added to fluorescently label EdU. The nuclei are stained with Hoechst for 15 min. Finally, DFs were viewed and photographed under the fluorescence microscope.

### Scratch assay

To assess the cell migration of DFs and HaCaT cells, the scratch assay was performed by scratching with the tip of a 10 µL pistol. After different treatments, the cells and the wound healing status were observed and photographed at 24 h, 36–72 h under a microscope. Using Image J software, we measured the migrated area.

### Measurement of intracellular ROS

The level of intracellular ROS was examined by the ROS assay kit (Nanjing Jiancheng Bioengineering Institute, E004-1-1). Briefly, cells with different treatments were seeded in six-well plates. After the medium was removed, cells were rinsed using 37°C pre-warmed PBS and incubated with 1 mL of 10 µM DCFH‐DA (1:1000, no serum) for 20 min at 37°C. The dye solution was washed away, and cells were washed using 37°C DMEM without serum three times. The stained ROS signals were then photographed using a fluorescent microscope and the absorbance was measured at 485 nm by microplate reader.

### Lipid peroxidation assay

Malondialdehyde (MDA) assay kits (Beyotime, China, S0131S) were used in accordance with the manufacturer’s guidelines to measure the level of MDA in aging dermal fibroblast (DFs) and HaCaT cells. Aging cells were seeded at 1 × 10^5^ cells per well into 12-well plates and incubated with the indicated treatments in an incubator of 5% CO_2_ at 37°C. 30 µl of cell lysis buffer (Beyotime, China, P0013) was added to the cells and incubated for 10 min. The cells were then detached using a cell scraper and transferred to a 1.5 mL tube along with the buffer. The tubes were vortexed every 10 min, and this process was repeated three times. The supernatant lysate from each tube was collected by centrifugation at 12,000 g, 4°C for 10 min. Subsequently, 100 µl of the lysate sample was mixed with 200 µl of malondialdehyde solution and incubated for 15 min at 100°C, protected from light. After cooling to room temperature, the mixtures were centrifuged at 1000 g, 25°C for 10 min. Next, 200 µl of the supernatant from each tube was transferred to a 96-well plate and immediately measured for absorbance at OD 532 nm. Additionally, a standard curve was simultaneously performed according to the manufacturer’s instructions.

### In vitro co-culture assay

DFs and HaCaT cells were co-cultured by indirect transwell co-culture. 4 × 10^5^ DFs were seeded in the upper compartment and treated with PBS, EVs^NC^ and EVs^*Gstm2*^ for 48 h. Then, the supernatant was replaced with fresh medium and 4 × 10^5^ HaCaT cells were seeded in the lower compartment of a transwell membrane. Then, the cells were incubated for another 48 h.

### Small interference RNA transfection

Small interfering RNA (siRNA) was purchased from Genomeditech (Shanghai) Co.,Ltd. The HaCaT cells were transfected with *NACA* siRNA (si*NACA*) (50 nM) or negative control siRNA (si-NC) (50nM) in mediation of Lipofectamine™ 2000 Transfection Reagent (Invitrogen Inc., Carlsbad, CA, USA). HaCaT cells in each group were seeded in a 12-well plate and cultured in an incubator at 37°C with 5% CO_2_ until 60% confluence. According to the operation manual of Lipofectamine 2000 Transfection Reagent, cell transfection was performed. The knockdown efficiency was confirmed at 48 h post transfection by qRT-PCR.

### Animals

All animal procedures were approved by the institutional Animal Care Committee and Use Committee of the Tongji University for Laboratory Animal Medicine (TJBB06123101). Three young male ICR mice (1-month old, body weight 20 ± 2 g) and 33 aging male ICR mice (12-month-old, body weight 50 ± 5 g) were provided by Shanghai Laboratory Animal Research Center (Shanghai, China). All mice were housed in plastic cages on a 12 h light/dark cycle and were allowed to drink water and eat freely.

### In vivo wound healing experiment

In order to detect the effect of EVs^*Gstm2*^ on wound healing [59], Treatment: 9 mice were randomly selected from 18 aging male ICR mice and were divided into three groups randomly: (a) 3 ICR mice treated with PBS (NC group); (b) 3 ICR mice treated with EVs^NC^ (EVs^NC^ group); (c) 3 ICR mice treated with EVs^*Gstm2*^ (EVs^*Gstm2*^ group). These mice were anaesthetized with an intraperitoneal injection of 2% pentobarbital sodium (60 mg/kg), shaved the hair with an electric razor, and a depilatory cream on the backs. The skin was sterilized with betadine and 70% alcohol. Then, full-thickness wounds were prepared on the back using a 6-mm biopsy punch. Immediately, the wound area was surrounded by a plasticine ring (inner diameter: 6 mm; height: 2 mm), which was then imaged using a digital camera. Mice in each group were administered subcutaneously PBS, EVs^NC^ and EVs^*Gstm2*^ at day 0, 4, 8 and 12, the wound area was observed and photographed to evaluate the wound healing simultaneously. The wound area and healed wound percentage were measured with Image J software, and wound healing were simulated with photoshop and Image J software (version 1.46r, USA).

### In vivo natural aging and treatment

12-month-old mice were used for the natural aging model. 9 mice (*n* = 9) were randomly selected from 18 aging male ICR mice and were divided into three groups randomly. Treatment: 9 ICR mice were randomly divided into three groups of five mice each: (a) 3 ICR mice treated with PBS; (b) 3 ICR mice treated with EVs^NC^; (c) 3 ICR mice treated with EVs^*Gstm2*^. In this experiment, PBS, EVs^NC^ and EVs^*Gstm2*^ were delivered by mini electric nano mesotherapy at days 0, 4, 8, 12. Needleless dermopressurs injector-EVs delivery consisted of one-time injections in 4 different sites evenly on the whole dorsal skin.

### Histological analysis

The histological analysis of the skin condition of each group of mice was assessed by H&E and Masson staining. Briefly, mouse skin tissues were embedded in paraffin and horizontally cut into 8 μm slices. According to the manufacturer’s protocol, these sections were stained with H&E and Masson trichrome after deparaffinization with xylene and hydration [60].

### Immunohistochemistry (IHC) analysis

IHC analysis was performed with standard procedures described previously [61]. According to manufacturers’ instructions, the following antibodies were used at indicated dilutions: primary Collagen I antibodies (1:200, Abclonal, A1352) and primary MMP2 antibodies (1:200, Servicebio, GB11130).

### Immunofluorescence staining

As previously described, immunofluorescence staining was performed with 4% PFA-fixed DFs and PFA-fixed paraffin-embedded tissue slices [62]. PFA-fixed paraffin-embedded tissue slices as previously described. Primary antibodies were: Vimentin (1:200, Abcam, ab92547), GSTM2 (1:200, Abclonal, A13496), and CD31(1:200, Abclonal, A0378, 1:300). All Alexa Fluor secondary antibodies (Molecular Probes) were used at dilutions of 1:200. Samples were counterstained with ProLong Gold anti-fading containing DAPI to label cell nuclei. Afterward, all stained cells were examined and photographed using fluorescent microscopy. The quantification of CD31 staining was conducted by a blinded manner, where the observer was unaware of the experimental conditions. At least five random images were captured for each experimental sample. The percentage of CD31-positive area was calculated using Image J software, and the mean percentage of CD31-positive area was determined for each field of view (*n* = 5).

### RNA sequencing (RNA-seq)

The raw data of RNA-seq were trimmed to remove adapters and low-quality reads through Trim Galore! (version 0.6.4_dev). Hisat2 (version 2.2.1) was used to align the clean reads to the mouse genome (mm10). FeatureCounts (version 2.0.1) was then applied to quantify the reads mapped to the genes. DEseq2 (version 1.28.1) was used to do the differential expression analysis. Genes with a fold change (FC) > 1 and adjusted *P*-value < 0.05 were considered as differentially expressed genes (DEGs). Gene Set Enrichment Analysis (GSEA) was conducted by clusterProfiler (version 3.16.1) R package.

### Statistical analysis

All data were analyzed with GraphPad Prism (version 8.3.0, USA) and calculated as mean ± standard deviation (mean ± SD). Statistical analyses were conducted using the student’s t-test or Analysis of Variance (ANOVA). Three biological replicates were performed for each experiment. Statistical significance was determined by a *p*-value less than 0.05.

### Electronic supplementary material

Below is the link to the electronic supplementary material.

Supplementary Material 1

## Data Availability

The raw sequence data reported in this paper have been deposited in the Genome Sequence Archive in National Genomics Data Center, China National Center for Bioinformation / Beijing Institute of Genomics, Chinese Academy of Sciences (GSA: CRA014960) that are publicly accessible at https://ngdc.cncb.ac.cn/gsa/browse/CRA014960 [63, 64]. The datasets used and/or analyzed during the current study are available from the corresponding author on reasonable request.
