# Supplementary material for: Improving dermal fibroblast-to-epidermis communications and aging wound repair through extracellular vesicle-mediated delivery of Gstm2 mRNA
Source: J Nanobiotechnology. 2024 Jun 2;22:307. doi: 10.1186/s12951-024-02541-1 (PMC11145791; doi:10.1186/s12951-024-02541-1)
Supplement: Supplementary file 1 — Supplementary Material 1 [file 12951_2024_2541_MOESM1_ESM.docx]

## Supporting information

**Improving Dermal Fibroblast-to-Epidermis Communications and Aging Wound Repair through Extracellular Vesicle-Mediated Delivery of *Gstm2* mRNA**

Haiyan Wu^1†^, Zuochao Yao^2†^, Hongkun Li^3†^, Laihai Zhang^4†^, Yuying Zhao^1^, Yongwei Li^5^, Yating Wu^1^, Zhenchun Zhang^1^, Jiali Xie^6^, Feixue Ding^7^ and Hongming Zhu^1*^

^1^ *Institute for Regenerative Medicine & Research Center for Translational Medicine, Shanghai East Hospital, Tongji University School of Medicine, Shanghai, 200120, China.*

^2^ *Department of Plastic and Reconstructive Surgery, Shanghai East Hospital, School of Medicine, Tongji University, Shanghai, 200120, China.*

^3^ *Department of Cardiology, Changzhi Medical College Affiliated Heji Hospital, Shanxi, 046000, China.*

^4^ *Department of Cardiothoracic Surgery, Shanghai East Hospital, School of Medicine, Tongji University, Shanghai 200092, China.*

^5^ Department of Cardiology, Shanghai East Hospital, School of Medicine, Tongji University, Shanghai 200092, China.

^6^ *Department of Neurology, Shanghai East Hospital, School of Medicine, Tongji University, Shanghai 200092, China.*

^7^ *Department of Plastic and Reconstructive Surgery, Shanghai Ninth People Hospital, School of Medicine, JiaoTong University, Shanghai 200001, China.*

†Haiyan Wu, Zuochao Yao, Hongkun Li and Laihai Zhang contributed equally to this work.

*Correspondence:

Hongming Zhu

[zhm@tongji.edu.cn](mailto:zhm@tongji.edu.cn)


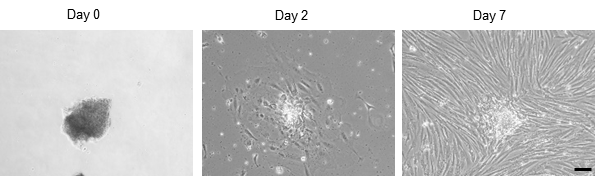


**Figure S1.** The migratory behavior of fibroblasts derived from the skin tissue of suckling mice was examined at day 0, day 2, to day 7. The scale bar is 50 μm.


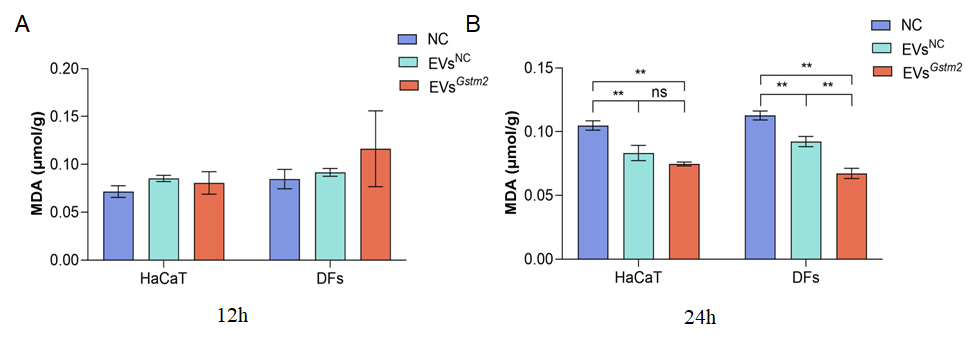


**Figure. S2**. **lipid peroxidation assay** (A-B) lipid peroxidation assay detects the expression levels of aging in Hacat and DFs cells at 12 and 24 hours post EVs treatment (mean ± SEM, n = 3). **P* < 0.01.


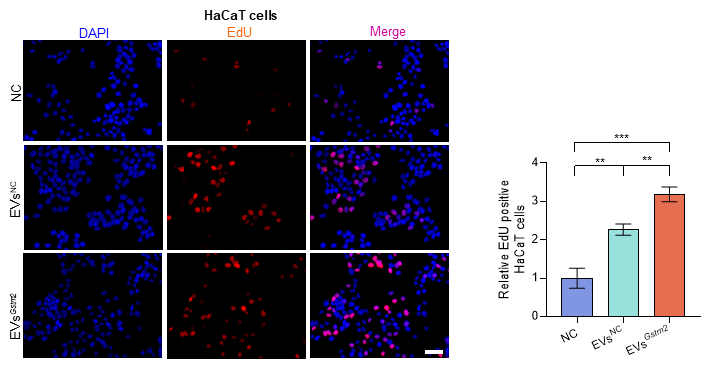


**Figure. S3.** EdU staining and quantification for HaCaT cells in NC group, EVs^NC^ group and EVs*^Gstm2^* group. The scale bar is 50 μm.


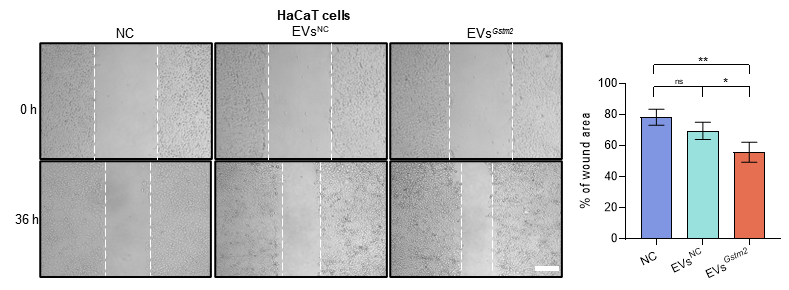


**Figure. S4.** Scratch wound healing assay and wound area statistics for HaCaT cells treated with PBS, EVs^NC^ and EVs*^Gstm2^* at 0 h and 36 h*.* The scale bar is 200 μm.


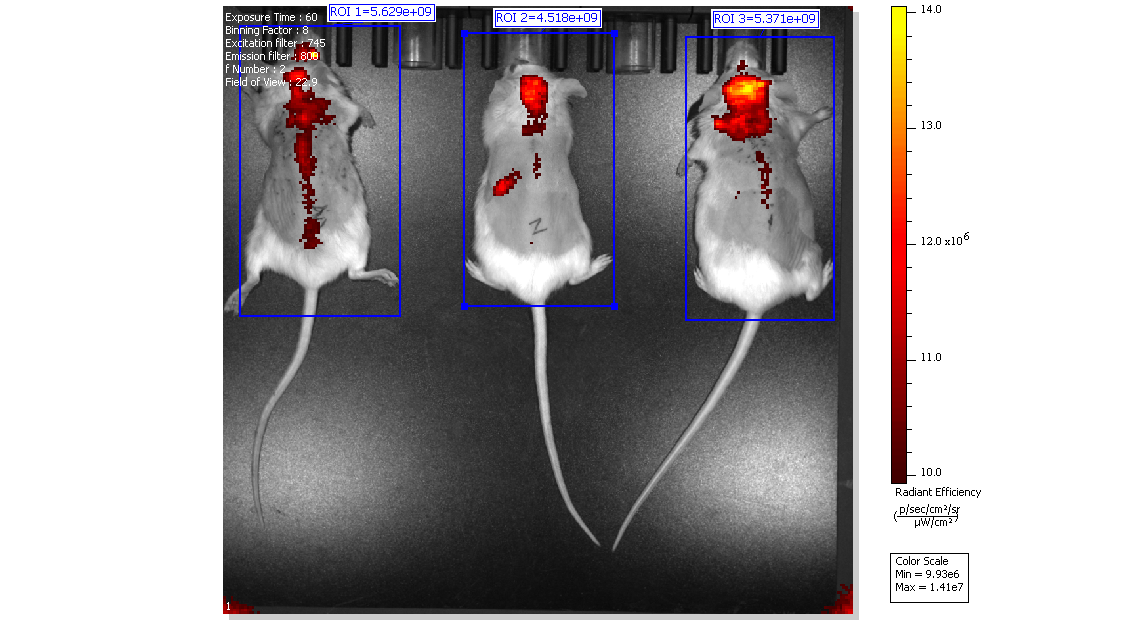


**Figure. S5.** In vivo small animal imaging. Dil-labeled EVs (2mL 100μg/mL) were injected into the back of mice by needle free injection, microneedle injection and subcutaneous injection, respectively. The mice fluorescence radiant efficiency was measured after 6h using the IVIS Spectrum. Left: needle free injection; middle: microneedle injection; right: subcutaneous injection.


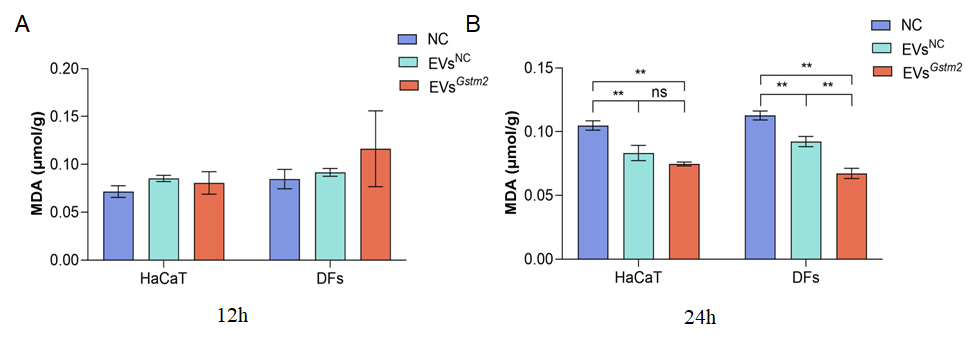


**Figure. S6.** mRNA expression of senescence-related gene prognostic index (*Csf3*, *Cxcl9*, *Cxcl12* and *Il17*).


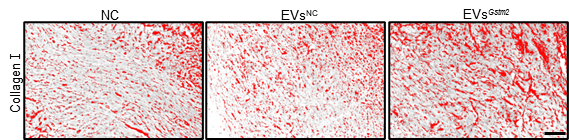


**Figure. S7.** Image J software was used to evaluate the expression of collagen Ⅰ. The scale bar is 100 μm.


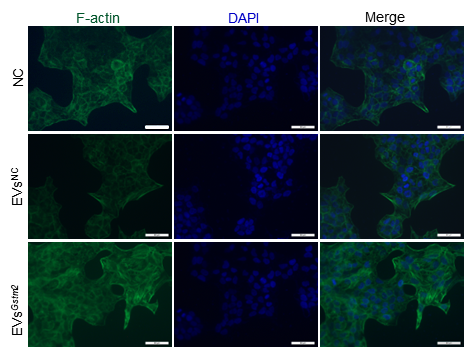


**Figure. S8.** Fluorescence microscopy analysis of F-actin organization in HaCaT cells. The scale bar is 50 μm.

**Supplementary Table 1. Characteristics of the young and old volunteers.**

| **Patient characteristic** |  |
| --- | --- |
| Young volunteers | n=3 |
| Age (years, mean ± SD) | 21 ± 2.16 |
| Sex (Male/Female) | 0/3 |
| Heart rate, per minute (mean ± SD) | 88 ± 2.16 |
| SBP, mmHG (mean ± SD) | 108.67 ± 1.25 |
| DBP, mmHG (mean ± SD) | 77 ± 1.63 |
| Hypertension, no. (%) | 0 |
| Diabetes mellitus, no. (%) | 0 |
| Hyperlipidemia, no. (%) | 0 |
| Old volunteers | n=3 |
| Age (years, mean ± SD) | 53.67 ± 3.30 |
| Sex (Male/Female) | 0/3 |
| Heart rate, per minute (mean ± SD) | 70 ± 4.08 |
| SBP, mmHG (mean ± SD) | 119.33 ± 3.30 |
| DBP, mmHG (mean ± SD) | 85 ± 1.63 |
| Hypertension, no. (%) | 0 |
| Diabetes mellitus, no. (%) | 0 |
| Hyperlipidemia, no. (%) | 0 |

**Supplementary Table 2. Sequence of the primers used in this study.**

| Genes | Sequence (5' to 3') |
| --- | --- |
| mouse *Tp53* F | GGCTGAGACACAATCCTCCC |
| mouse *Tp53* R | CATTGTAGGTGCCAGGGTCC |
| mouse *Cdkn2a* F | ATTGTGGGTCTAGGGTGGGT |
| mouse *Cdkn2a* R | AGTGTCCTGTGAGCTCCCTT |
| mouse *Cdkn1a* F | CCAAGAGCGGGGACATCAAG |
| mouse *Cdkn1a* R | CTCTGCTCTTGGGATTGGCC |
| mouse *Mmp3* F | GGCGCAAATCTCTCAGGACT |
| mouse *Mmp3* R | TGCCCTCGTATAGCCCAGAA |
| mouse *Gstm2* F | TTCTGTGATGGGCTGTGTCC |
| mouse G*stm2* R | CACACAGGTTGTGCTTTCGG |
| mouse *Col1a1* F | GCTCCTCTTAGGGGCCACT |
| mouse *Col1a1* R | ATTGGGGACCCTTAGGCCAT |
| mouse *Ndufaf8* F | CTGTCCTGACAGCACGCATA |
| mouse *Ndufaf8* R | TCCTCATGTACACACTGCCG |
| mouse *Ndufaf4* F | CACCGGAGTCAGTATCCAGAA |
| mouse *Ndufaf4* R | GGTTCAACTTTTACCGGCAAGG |
| mouse *Ndufa12* F | GGCCTCCTACGGGTTTTCTTC |
| mouse *Ndufa12* R | GGTGGTGTAGATGACCCATCG |
| mouse *Ndufb4c* F | TTTTAGGAGCTGTGGCAGGG |
| mouse *Ndufb4c* R | TCTTGCCAAACTTAGTAGGAGATGT |
| mouse *Ndufv2* F | GCAAGGAATTTGCATAAGACAGC |
| mouse *Ndufv2* R | TAGCCATCCATTCTGCCTTTG |
| mouse *Ndufaf2* F | CACGGACCATCTGGGGAAC |
| mouse *Ndufaf2* R | CTTTTCTCTCGAATAGTCTGCCC |
| mouse *Ndufa4* F | TCCCAGCTTGATTCCTCTCTT |
| mouse *Ndufa4* R | GGGTTGTTCTTTCTGTCCCAG |
| mouse *Ndufb4* F | CGGGTTCCAAGTATAAGCCTG |
| mouse *Ndufb4* R | GTTTGGGGTCGTTGTACTGAAG |
| mouse *Ndufs8* F | GTGGCGGCAACGTACAAGTAT |
| mouse *Ndufs8* R | GAATCCGAGCTGCATTGTCAG |
| mouse *Cox7a2* F | GCTGGCCCTTCGTCAGATT |
| mouse *Cox7a2* R | GGCATCCCATTATCCTCCTGAA |
| mouse *Gapdh* F | GTCATCCCAGAGCTGAACGG |
| mouse *Gapdh* R | CTTCAGTGGGCCCTCAGATG |
| mouse *Actin* F | GACATGCCCTCATTCCACCA |
| mouse *Actin* R | GAGAGAGAGAGAGCGAGCCT |
| mouse *Csf3* F | GCACTATGGTCAGGACGAGAG |
| mouse *Csf3* R | GGGGAAATACCCGATAGAGCC |
| mouse *Cxcl9* F | GGAGTTCGAGGAACCCTAGTG |
| mouse *Cxcl9* R | GGGATTTGTAGTGGATCGTGC |
| mouse *CXCL12* F | TGCATCAGTGACGGTAAACCA |
| mouse *CXCL12* R | CACAGTTTGGAGTGTTGAGGAT |
| mouse *Il-7* F | TTCCTCCACTGATCCTTGTTCT |
| mouse *Il-7* R | AGCAGCTTCCTTTGTATCATCAC |
| mouse *Vegf* F | CTGCCGTCCGATTGAGACC |
| mouse *Vegf* R | CCCCTCCTTGTACCACTGTC |
| mouse *Igf-1* F | CACATCATGTCGTCTTCACACC |
| mouse *Igf-1* R | GGAAGCAACACTCATCCACAATG |
| human *GAPDH* F | GGAGCGAGATCCCTCCAAAAT |
| human *GAPDH* R | GGCTGTTGTCATACTTCTCATGG |
| human *ETS* F | GATAGTTGTGATCGCCTCACC |
| human *ETS* R | GTCCTCTGAGTCGAAGCTGTC |
| human *CCNDI* F | GCTGCGAAGTGGAAACCATC |
| human *CCNDI* R | CCTCCTTCTGCACACATTTGAA |
| human *GSTM2* F | TGTGCGGGGAATCAGAAAAGG |
| human *GSTM2* R | CTGGGTCATAGCAGAGTTTGG |
| human *NACA* F | TGGAACAGAATCTGACAGTGATG |
| human *NACA* R | CTGGGCTTGTTGTGTGGTTG |
